# Genome-wide identification, characterisation and expression profiling of the ubiquitin-proteasome genes in *Biomphalaria glabrata*

**DOI:** 10.1590/0074-02760190052

**Published:** 2019-06-03

**Authors:** Laysa Gomes Portilho, Bruna Custódio Dias Duarte, Fábio Ribeiro Queiroz, Thales Henrique Cherubino Ribeiro, Wander de Jesus Jeremias, Elio Hideo Babá, Paulo Marcos Zech Coelho, Enyara Rezende Morais, Fernanda Janku Cabral, Roberta Lima Caldeira, Matheus de Souza Gomes

**Affiliations:** 1 Universidade Federal de Uberlândia, Laboratório de Bioinformática e Análises Moleculares, Patos de Minas, MG, Brasil; 2 Fundação Oswaldo Cruz-Fiocruz, Instituto René Rachou, Grupo de Pesquisa em Biologia do Schistosoma mansoni e sua Interação com o Hospedeiro, Belo Horizonte, MG, Brasil; 3 Universidade Federal de Lavras, Departamento de Biologia, Seção de Fisiologia de Plantas, Laboratório de Fisiologia Molecular de Plantas, Lavras, MG, Brasil; 4 Universidade Federal de Ouro Preto, Departamento de Farmácia/Escola de Farmácia, Ouro Preto, MG, Brasil; 5 Universidade Federal de Uberlândia, Laboratório de Bioquímica e Biologia Molecular, Patos de Minas, MG, Brasil; 6 Fundação Oswaldo Cruz-Fiocruz, Instituto René Rachou, Grupo de Pesquisa em Helmintologia e Malacologia Médica, Belo Horizonte, MG, Brasil; 7 Universidade Estadual de Campinas, Instituto de Biologia, Departamento de Biologia Animal, Campinas, SP, Brasil

**Keywords:** snail, UPS, signaling pathway, bioinformatics, schistosomiasis

## Abstract

**BACKGROUND:**

*Biomphalaria glabrata* is the major species used for the study of schistosomiasis-related parasite-host relationships, and understanding its gene regulation may aid in this endeavor. The ubiquitin-proteasome system (UPS) performs post-translational regulation in order to maintain cellular protein homeostasis and is related to several mechanisms, including immune responses.

**OBJECTIVE:**

The aims of this work were to identify and characterise the putative genes and proteins involved in UPS using bioinformatic tools and also their expression on different tissues of *B. glabrata*.

**METHODS:**

The putative genes and proteins of UPS in *B. glabrata* were predicted using BLASTp and as queries reference proteins from model organism. We characterised these putative proteins using PFAM and CDD software describing the conserved domains and active sites. The phylogenetic analysis was performed using ClustalX2 and MEGA5.2. Expression evaluation was performed from 12 snail tissues using RPKM.

**FINDINGS:**

119 sequences involved in the UPS in *B. glabrata* were identified, which 86 have been related to the ubiquitination pathway and 33 to proteasome. In addition, the conserved domains found were associated with the ubiquitin family, UQ_con, HECT, U-box and proteasome. The main active sites were lysine and cysteine residues. Lysines are responsible and the starting point for the formation of polyubiquitin chains, while the cysteine residues of the enzymes are responsible for binding to ubiquitin. The phylogenetic analysis showed an organised distribution between the organisms and the clades of the sequences, corresponding to the tree of life of the animals, for all groups of sequences analysed. The ubiquitin sequence was the only one with a high expression profile found in all libraries, inferring its wide range of performance.

**MAIN CONCLUSIONS:**

Our results show the presence, conservation and expression profile of the UPS in this mollusk, providing a basis and new knowledge for other studies involving this system. Due to the importance of the UPS and *B. glabrata*, this work may influence the search for new methodologies for the control of schistosomiasis.

*Biomphalaria glabrata* is a species of snail that has considerable epidemiological importance, since their presence represents a crucial condition for the dissemination of schistosomiasis, affecting more than 200 million people around the word. These mollusks are considered intermediate hosts of *Schistosoma mansoni* and present a high degree of susceptibility to helminth infection. In addition, they have a wide geographical distribution in the Americas.^1^ ^,^ ^2^ Due to the importance of this organism, many studies seek new knowledge about its biology. It is already known that the interaction between mollusk and trematode is complex, and the expression of genes involved in host susceptibility/resistance and parasite infectivity is fairly well-understood.^3^ Thus, one of the ways to better understand this relationship is to deepen studies related to gene and protein regulation in *B. glabrata*, because the infection carried out by *S. mansoni* leads to changes in the expression profile of some proteins and, consequently, in the defense pattern of the mollusk.^4^ ^,^ ^5^ Thus, it is necessary to evaluate the regulatory systems of expression in these organisms, on the gene and protein level. One of the key components in protein regulation is the ubiquitin-proteasome system, as it performs specific post-translational regulation and assists in the maintenance of protein homeostasis in cells.

Given the epidemiological importance of *B. glabrata*, Adema and contributors,^6^ including our working group, published a complete analysis of the snail genome. Genes involved in communication with the aquatic environment, stress, innate immunity and regulation of biological processes were identified and described, as well as several small RNAs related to gene regulation. In addition, transcripts involved in the ubiquitin-proteasome system were found, from genes encoding enzymes to accessory proteins and the subunits that form the proteolytic 26S proteasome.^6^

The ubiquitin-proteasome system (UPS) is composed of two main elements: the ubiquitination pathway and the proteolytic macromolecule 26S proteasome. First, the ubiquitination pathway labels proteins with one or more ubiquitin molecules that are then degraded by the 26S proteasome.^7^ This system is able to degrade mutated and defective proteins that are involved in important cellular processes, such as cell cycle regulation, stress response and extracellular modulators, DNA repair and the regulation of immune system and inflammatory responses.^8^ ^,^ ^9^

The first class acting on the ubiquitination pathway are ubiquitin-activating enzymes (E1) that activate the ubiquitin molecule in an ATP-dependent manner and generate an ubiquitin E1-thioester. Subsequently, ubiquitin conjugating enzymes (E2) catalyse the covalent binding reaction of activated ubiquitin with target protein substrate. At the end of this cascade, the enzymes ubiquitin ligases (E3) recognise the specific protein to be degraded and assist in the transfer of the ubiquitin present in E2 to the substrate, being able to bind both E2-ubiquitin and target substrate concomitantly or at different times.^7^ ^,^ ^10^ At the end of the first step, the labeled substrates are degraded by the proteolytic complex 26S proteasome. This protease is formed from an association between a 19S (PA700) regulatory particle divided between lid and base which are reversibly connected and ATP-dependent to the central component 20S.^7^ ^,^ ^11^ The regulatory particle recognises, unfolds and translocates the substrate to the central particle.^12^ However, the central component 20S has proteolytic sites that play the role of degrading the target protein, since they have similar functions to caspase-like, chymotrypsin-like and trypsin-like.^13^ Soon after, the target proteins are degraded into smaller peptides and the ubiquitin molecules present in the tail are released.^7^

The peptides generated from the digestion performed by the proteasome are related to immunity. In humans, these peptides are recognised as epitopes by MHC class I and, thus, the proteasome plays an essential role in this recognition.^14^ However, the immunoproteasome, a standard isoform of the proteasome, is directly involved with the immune system because it is more efficient for the generation of antigenic peptides. This isoform has three different subunits in the 20S nucleus as compared to the proteasome 26S, β1i (LMP2), β2i (MECL-1) and β5i (LMP7), respectively. They substitute constitutive subunits and are therefore assembled more rapidly, triggering a more agile immune response in hematopoietic cells, in addition to modifying peptidase activity and increasing epitope generation.^15^ In addition, the thymoproteasome is another isoform specifically expressed in the thymus; both are involved in cell-mediated immunity.^16^ Thus, the relationship involving the catalytic proteasome and immunity has led us to believe that it is also related to the resistance/susceptibility that some organisms present when they are infected by pathogens.

Genes involved in the ubiquitination pathway and in proteasome formation have already been reported in studies involving the cells of the internal defense system, i.e. hemocytes. These genes demonstrated a different expression profile when analysed in infected and uninfected snails.^17^ ^,^ ^18^ ^,^ ^19^ Thus, the hypothesis of this study is that the UPS has been conserved in the genome of *B. glabrata* and is expressed at the transcriptional level; that is, it is present in the transcriptome data in all the parts of the body of the uninfected adult snail. Thus, the aims of this work were to identify and characterise the UPS in the genome and transcriptome data of the snail using *in silico* analyses, as well as to characterise the classes involved in this system and to evaluate the expression profile of the sequences identified in different tissues of the adult snail.

## MATERIALS AND METHODS

*Identification of UPS genes in B. glabrata* - The genome and transcriptome data used for analysis *in silico* were retrieved from the last version of VectorBase database, i.e. the BglaB1 genome of *B. glabrata* (https://www.vectorbase.org/organisms/biomphalaria-glabrata). Initially, the prediction of genes involved in the ubiquitin-proteasome pathway was performed through bibliographic searches and KEGG (https://www.genome.jp/kegg/pathway.html) in model organisms such as *Drosophila melanogaster* and *Caenorhabditis elegans*. The sequences belonging to the model organisms were obtained from the NCBI database (https://www.ncbi.nlm.nih.gov/) and later used as a queries against the genome and transcriptome of the snail, seeking to find UPS genes. Subsequently, the sequences identified in *B. glabrata* data were grouped according to the role they play and also analysed for their conserved domains, active sites and gene expression.

*Sequence analysis* - Sequences were fed into the protein family database, PFAM (https://pfam.xfam.org/), to identify the major conserved characteristic domains belonging to each previously organised sequence group. CDD (https://www.ncbi.nlm.nih.gov/Structure/cdd/wrpsb.cgi) was used to search for amino acid residues involved in active site formations, structural motifs and catalytic clefts.

*Phylogenetic analysis* - To determine the evolutionary organisation and distribution of the sequences found, as well as to provide further evidence of the presence of probable proteins found in the snail, phylogenetic analysis was performed using MEGA5.2. For the analysis, amino acid sequences of deuterostomes and protostomes organisms such as *Homo sapiens*, *Mus musculus*, *D. melanogaster*, *C. elegans* and mollusks such as *Aplysia californica* and *Lottia gigantea*, obtained from NCBI were used. ClustalX 2.1 was used to perform multiple sequence alignment and their output fed in the MEGA 5.2 software, using the neighbor-joining method and JTT model for calculations of evolutionary distance. The consensus tree was inferred from a bootstrap of 1000 replicates to represent the evolutionary history of the study.^20^

*Expression analysis* - For the expression analysis, all transcripts identified in the *B. glabrata* genome data were individually submitted and analysed against an RNASeq data set for a library of 12 different snail tissues [Supplementary data (Table)].^6^ Quality control and adapter removal were conducted using Trimmomatic (v 0.36). Single-end reads were aligned against pre-selected sequences using bowtie2 (v 2.3.0) with the “-very-sensitive-local parameter”. Alignment sam files were sorted and converted to bam files with samtools (v 1.6). Expression values were extracted from the alignment results using express (v 1.5.1) and RPKM (Reads Per Kilobase of transcript, per Million mapped reads) were calculated after library size normalisation with the Bioconductor package edgeR5 in the R statistical environment. The expression profile was plotted so that the more intense the red color, the more expressed the gene was presented to the corresponding library; a more intense green color indicates less expression found in the data used in the analysis.

## RESULTS

*Identification and characterisation of putative proteins of the UPS complex: alignment and phylogenetic analysis* - In total, 119 sequences were found in the genome, transcriptome and predicted proteome of *B. glabrata* involved in the UPS. Among them, 86 were found to be involved with the ubiquitination pathway and 33 with 26S proteasome formation. Among the 86 genes of the ubiquitination pathway, one ubiquitin gene, six E1, 22 E2 and 39 E3 were identified, divided into the HECT, RING finger and U-box groups. In addition, 19 genes were found to be related to the forming of 26S proteasome and involved with the regulatory portion, while 14 involved in form the nucleus of the molecule (Table I). The genes were grouped according to their characteristics and functions based on the analysis of conserved domains, active sites and phylogenetic analysis.

**TABLE I:** Genes involved in ubiquitin-proteasome system (UPS) identified in *Biomphalaria glabrata* transcriptome and their orthologous organism

Putative gene name	Function	ID *B. glabrata* NCBI	Predicted size (aa)	ID Ortholog NCBI	Ortholog specie	Size (aa) Ortholog	E-value (Blastp)
*ubq-1*	Ubiquitin	BGLB020284-PA	229 aa	NP_741158.2	*Caenorhabditis elegans*	538 aa	1^-156^
*uba1-a*	E1	BGLB013827-PB	1029 aa	NP_001033404.1	*Caenorhabditis elegans*	1113 aa	0.0
*uba1-b*	E1	BGLB011911-PC	1014 aa	NP_001033405.1	*Caenorhabditis elegans*	1028 aa	0.0
*uba1-c*	E1	BGLB011911-PB	1015 aa	NP_001255449.1	*Caenorhabditis elegans*	1112 aa	0.0
*aos-1*	E1	BGLB007929-PB	339 aa	NP_505604.1	*Caenorhabditis elegans*	343 aa	6^-38^
*uba2*	E1	BGLB013435-PB	626 aa	NP_001293154.1	*Caenorhabditis elegans*	582 aa	2^-171^
*rfl-1*	E1	BGLB035057-PA	347 aa	NP_498534.2	*Caenorhabditis elegans*	430 aa	2^-58^
*ubc1-a*	E2	BGLB000489-PB	172 aa	NP_500480.1	*Caenorhabditis elegans*	192 aa	8^-97^
*ubc1-b*	E2	BGLB000489-PC	156 aa	NP_500480.1	*Caenorhabditis elegans*	192 aa	3^-100^
*ubc2-a*	E2	BGLB005575-PB	147 aa	NP_502065.1	*Caenorhabditis elegans*	147 aa	4^-94^
*ubc2-b*	E2	BGLB005576-PB	147 aa	NP_502065.1	*Caenorhabditis elegans*	147 aa	2^-93^
*ubc2-c*	E2	BGLB000510-PC	147 aa	NP_502065.1	*Caenorhabditis elegans*	147 aa	8^-90^
*ubc2-d*	E2	BGLB000510-PB	147 aa	NP_502065.1	*Caenorhabditis elegans*	147 aa	8^-90^
*ubc-3*	E2	BGLB003582-PB	236 aa	NP_490882.3	*Caenorhabditis elegans*	243 aa	6^-95^
*ubc-6*	E2	BGLB000513-PB	660 aa	NP_001040755.1	*Caenorhabditis elegans*	314 aa	1^-79^
*ubc-7*	E2	BGLB008113-PB	167 aa	NP_499133.1	*Caenorhabditis elegans*	164 aa	1^-88^
*ubc-8*	E2	BGLB025873-PA	181 aa	NP_500245.2	*Caenorhabditis elegans*	216 aa	8^-84^
*ubc-9-a*	E2	BGLB002089-PC	161 aa	NP_001023158.1	*Caenorhabditis elegans*	166 aa	3^-96^
*ubc-9-b*	E2	BGLB002089-PB	161 aa	NP_001023158.1	*Caenorhabditis elegans*	166 aa	3^-96^
*ubc-12-a*	E2	BGLB010485-PC	179 aa	NP_493024.1	*Caenorhabditis elegans*	180 aa	5^-55^
*ubc-12-b*	E2	BGLB010485-PB	179 aa	NP_493024.1	*Caenorhabditis elegans*	180 aa	5^-55^
*ubc-13-a*	E2	BGLB033527-PB	96 aa	NP_500272.2	*Caenorhabditis elegans*	151 aa	2^-55^
*ubc-13-b*	E2	BGLB033527-PA	96 aa	NP_500272.2	*Caenorhabditis elegans*	151 aa	2^-55^
*ubc-14*	E2	BGLB011939-PB	165 aa	NP_493381.1	*Caenorhabditis elegans*	170 aa	5^-96^
*ubc-16*	E2	BGLB002705-PB	151 aa	NP_493587.1	*Caenorhabditis elegans*	152 aa	1^-60^
*ubc-18*	E2	BGLB002910-PB	157 aa	NP_498541.1	*Caenorhabditis elegans*	153 aa	7^-74^
*ubc-20*	E2	BGLB003190-PB	199 aa	NP_497174.1	*Caenorhabditis elegans*	199 aa	1^-82^
*ubc-25*	E2	BGLB036058-PA	366 aa	NP_492764.2	*Caenorhabditis elegans*	387 aa	8^-124^
*ubc-26*	E2	BGLB007384-PB	244 aa	NP_001337290.1	*Caenorhabditis elegans*	228 aa	4^-54^
*etc-1*	E3 (HECT)	BGLB018372-PA	1088 aa	NP_495842.1	*Caenorhabditis elegans*	1001 aa	1^-96^
*herc2*	E3 (HECT)	BGLB016413-PA	4933 aa	NP_608388.2	*Drosophila melanogaster*	4912 aa	0.0
*herc4-a*	E3 (HECT)	BGLB002480-PB	1046 aa	NP_728591.1	*Drosophila melanogaster*	1058 aa	0.0
*herc4-b*	E3 (HECT)	BGLB002480-PD	1047 aa	NP_728591.1	*Drosophila melanogaster*	1058 aa	0.0
*herc4-c*	E3 (HECT)	BGLB002480-PC	875 aa	NP_728591.1	*Drosophila melanogaster*	1058 aa	0.0
*oxi-1*	E3 (HECT)	BGLB002940-PB	1025 aa	NP_499392.1	*Caenorhabditis elegans*	1066 aa	0.0
*smurf-1-a*	E3 (HECT)	BGLB002099-PB	1023 aa	NP_523779.1	*Drosophila melanogaster*	1061 aa	0.0
*smurf-1-b*	E3 (HECT)	BGLB002099-PC	1024 aa	NP_523779.1	*Drosophila melanogaster*	1061 aa	0.0
*ube3a*	E3 (HECT)	BGLB000488-PB	902 aa	NP_648452.1	*Drosophila melanogaster*	973 aa	0.0
*wwp-1-a*	E3 (HECT)	BGLB008139-PC	842 aa	NP_740775.1	*Caenorhabditis elegans*	794 aa	0.0
*wwp-1-b*	E3 (HECT)	BGLB008139-PB	842 aa	NP_740775.1	*Caenorhabditis elegans*	794 aa	0.0
*Y92H12A.2*	E3 (HECT)	BGLB007073-PB	728 aa	NP_001293292.1	*Caenorhabditis elegans*	724 aa	2^-137^
*diap1*	E3 (RING finger)	BGLB013412-PB	507 aa	NP_524101.2	*Drosophila melanogaster*	438 aa	8^-56^
*gei-17-a*	E3 (RING finger)	BGLB038318-PA	578 aa	NP_001021677.3	*Caenorhabditis elegans*	663 aa	1^-60^
*gei-17-b*	E3 (RING finger)	BGLB038318-PE	682 aa	NP_001021677.3	*Caenorhabditis elegans*	663 aa	1^-60^
*gei-17-c*	E3 (RING finger)	BGLB038318-PB	713 aa	NP_001021677.3	*Caenorhabditis elegans*	663 aa	2^-60^
*gei-17-d*	E3 (RING finger)	BGLB038318-PD	714 aa	NP_001021677.3	*Caenorhabditis elegans*	663 aa	2^-60^
*gei-17-e*	E3 (RING finger)	BGLB038318-PC	714 aa	NP_001021677.3	*Caenorhabditis elegans*	663 aa	3^-46^
*sel-11*	E3 (RING finger)	BGLB003225-PB	683 aa	NP_505969.1	*Caenorhabditis elegans*	610 aa	2^-138^
*siah-1*	E3 (RING finger)	BGLB009524-PB	248 aa	NP_500409.1	*Caenorhabditis elegans*	419 aa	4^-111^
*sli-1*	E3 (RING finger)	BGLB017220-PA	659 aa	NP_001024798.1	*Caenorhabditis elegans*	523 aa	2^-154^
*traf6-a*	E3 (RING finger)	BGLB026988-PC	599 aa	NP_511080.2	*Drosophila melanogaster*	475 aa	1^-22^
*traf6-b*	E3 (RING finger)	BGLB026988-PB	599 aa	NP_511080.2	*Drosophila melanogaster*	475 aa	1^-22^
*traf6-c*	E3 (RING finger)	BGLB026988-PA	599 aa	NP_511080.2	*Drosophila melanogaster*	475 aa	1^-22^
*cg11070-a*	E3 (U-box)	BGLB035600-PD	1056 aa	NP_609060.1	*Drosophila melanogaster*	993 aa	0.0
*cg11070-b*	E3 (U-box)	BGLB035600-PB	1056 aa	NP_609060.1	*Drosophila melanogaster*	993 aa	0.0
*cg11070-c*	E3 (U-box)	BGLB035600-PA	1056 aa	NP_609060.1	*Drosophila melanogaster*	993 aa	0.0
*cg11070-d*	E3 (U-box)	BGLB035600-PC	1055 aa	NP_609060.1	*Drosophila melanogaster*	993 aa	0.0
*chn-1-a*	E3 (U-box)	BGLB001813-PI	275 aa	NP_491781.2	*Caenorhabditis elegans*	266 aa	2^-71^
*chn-1-b*	E3 (U-box)	BGLB001813-PH	275 aa	NP_491781.2	*Caenorhabditis elegans*	266 aa	2^-71^
*chn-1-c*	E3 (U-box)	BGLB001813-PG	275 aa	NP_491781.2	*Caenorhabditis elegans*	266 aa	2^-71^
*chn-1-d*	E3 (U-box)	BGLB001813-PF	275 aa	NP_491781.2	*Caenorhabditis elegans*	266 aa	2^-71^
*chn-1-e*	E3 (U-box)	BGLB001813-PE	275 aa	NP_491781.2	*Caenorhabditis elegans*	266 aa	2^-71^
*chn-1-f*	E3 (U-box)	BGLB001813-PD	275 aa	NP_491781.2	*Caenorhabditis elegans*	266 aa	2^-71^
*chn-1-g*	E3 (U-box)	BGLB001813-PC	275 aa	NP_491781.2	*Caenorhabditis elegans*	266 aa	2^-71^
*chn-1-h*	E3 (U-box)	BGLB001813-PB	275 aa	NP_491781.2	*Caenorhabditis elegans*	266 aa	2^-71^
*cyn-4*	E3 (U-box)	BGLB022189-PA	400 aa	NP_496337.1	*Caenorhabditis elegans*	523 aa	4^-140^
*prp-19*	E3 (U-box)	BGLB008873-PB	514 aa	NP_001293643.1	*Caenorhabditis elegans*	492 aa	0.0
*ufd-2*	E3 (U-box)	BGLB026024-PA	938 aa	NP_495692.1	*Caenorhabditis elegans*	980 aa	2^-60^
*apc-11*	Complexes (multi subunit RING finger)	BGLB005038-PB	91 aa	NP_497937.1	*Caenorhabditis elegans*	135 aa	6^-22^
*rbx-2*	Complexes (multi subunit RING finger)	BGLB034254-PA	101 aa	NP_491849.1	*Caenorhabditis elegans*	112 aa	5^-45^
*kaep-1*	Complexes (target recognizing subunit)	BGLB002604-PB	581 aa	NP_650594.1	*Drosophila melanogaster*	744 aa	0.0
*fizzy-a*	Complexes (target recognizing subunit)	BGLB002564-PD	525 aa	NP_477501.1	*Drosophila melanogaster*	526 aa	1^-175^
*fizzy-b*	Complexes (target recognizing subunit)	BGLB002564-PC	525 aa	NP_496075.1	*Caenorhabditis elegans*	702 aa	1^-98^
*fizzy-c*	Complexes (target recognizing subunit)	BGLB002564-PB	525 aa	NP_496075.1	*Caenorhabditis elegans*	702 aa	1^-98^
*fizzy-d*	Complexes (target recognizing subunit)	BGLB005726-PB	508 aa	NP_496075.1	*Caenorhabditis elegans*	702 aa	3^-170^
*fizzy-e*	Complexes (target recognizing subunit)	BGLB005726-PC	479 aa	NP_496075.1	*Caenorhabditis elegans*	702 aa	4^-170^
*sel-10*	Complexes (target recognizing subunit)	BGLB003995-PB	803 aa	NP_001023975.1	*Caenorhabditis elegans*	585 aa	2^-130^
*skpt-1*	Complexes (target recognizing subunit)	BGLB032982-PA	340 aa	NP_741136.1	*Caenorhabditis elegans*	418 aa	4^-42^
*skr-1*	Complexes (adapters)	BGLB010817-PB	162 aa	NP_492513.1	*Caenorhabditis elegans*	176 aa	1^-80^
*ddb-1*	Complexes (adapters)	BGLB013874-PB	1141 aa	NP_502299.1	*Caenorhabditis elegans*	1134 aa	0.0
*apc-2*	Complexes (accessories)	BGLB018106-PA	1024 aa	NP_498762.2	*Caenorhabditis elegans*	731 aa	8^-58^
*cul-1*	Complexes (accessories)	BGLB017332-PA	777 aa	NP_499309.1	*Caenorhabditis elegans*	780 aa	0.0
*cul-2*	Complexes (accessories)	BGLB030598-PA	769 aa	NP_001023008.1	*Caenorhabditis elegans*	791 aa	7^-175^
*cul-3*	Complexes (accessories)	BGLB002824-PB	736 aa	NP_503151.1	*Caenorhabditis elegans*	777 aa	0.0
*cul-4*	Complexes (accessories)	BGLB008547-PB	809 aa	NP_503151.1	*Caenorhabditis elegans*	840 aa	3^-130^
*cul-5*	Complexes (accessories)	BGLB004757-PB	777 aa	NP_505616.2	*Caenorhabditis elegans*	765 aa	0.0
*pas-1*	Core particles	BGLB011749-PB	246 aa	NP_506571.1	*Caenorhabditis elegans*	246 aa	8^-107^
*pas-2*	Core particles	BGLB007197-PB	260 aa	NP_505750.1	*Caenorhabditis elegans*	231 aa	3^-96^
*pas-3*	Core particles	BGLB014380-PB	242 aa	NP_491520.2	*Caenorhabditis elegans*	250 aa	1^-90^
*pas-4*	Core particles	BGLB005527-PB	249 aa	NP_492360.1	*Caenorhabditis elegans*	253 aa	1^-87^
*pas-5*	Core particles	BGLB016351-PA	278 aa	NP_492765.1	*Caenorhabditis elegans*	248 aa	2^-36^
*pas-6*	Core particles	BGLB006734-PB	278 aa	NP_504472.1	*Caenorhabditis elegans*	260 aa	9^-99^
*pas-7*	Core particles	BGLB010143-PC	254 aa	NP_496177.2	*Caenorhabditis elegans*	250 aa	1^-77^
*pbs-1*	Core particles	BGLB003737-PB	150 aa	NP_500125.1	*Caenorhabditis elegans*	239 aa	2^-43^
*pbs-2*	Core particles	BGLB002969-PB	274 aa	NP_493271.1	*Caenorhabditis elegans*	277 aa	1^-69^
*pbs-3*	Core particles	BGLB010370-PB	205 aa	NP_494913.1	*Caenorhabditis elegans*	204 aa	2^-73^
*pbs-4*	Core particles	BGLB008545-PB	168 aa	NP_491261.1	*Caenorhabditis elegans*	202 aa	2^-35^
*pbs-5*	Core particles	BGLB007975-PB	237 aa	NP_493558.1	*Caenorhabditis elegans*	284 aa	2^-70^
*pbs-6*	Core particles	BGLB002875-PB	222 aa	NP_498806.1	*Caenorhabditis elegans*	258 aa	2^-52^
*pbs-7*	Core particles	BGLB032647-PA	111 aa	NP_492354.1	*Caenorhabditis elegans*	236 aa	6^-21^
*rpn-1*	Regulatory particle	BGLB004929-PB	829 aa	NP_501064.1	*Caenorhabditis elegans*	981 aa	0.0
*rpn-2*	Regulatory particle	BGLB002581-PB	1005 aa	NP_498346.2	*Caenorhabditis elegans*	965 aa	0.0
*rpn-3*	Regulatory particle	BGLB004127-PB	500 aa	NP_498869.1	*Caenorhabditis elegans*	504 aa	3^-118^
*rpn-5*	Regulatory particle	BGLB001868-PB	458 aa	NP_494835.1	*Caenorhabditis elegans*	490 aa	4^-118^
*rpn-6.1*	Regulatory particle	BGLB008605-PB	419 aa	NP_001022621.1	*Caenorhabditis elegans*	438 aa	7^-157^
*rpn-7*	Regulatory particle	BGLB011257-PB	272 aa	NP_501632.1	*Caenorhabditis elegans*	410 aa	2^-99^
*rpn-8*	Regulatory particle	BGLB005296-PB	330 aa	NP_491319.1	*Caenorhabditis elegans*	362 aa	1^-132^
*rpn-9*	Regulatory particle	BGLB005529-PB	333 aa	NP_496405.1	*Caenorhabditis elegans*	387 aa	2^-66^
*rpn-10*	Regulatory particle	BGLB003999-PB	404 aa	NP_492809.1	*Caenorhabditis elegans*	346 aa	1^-97^
*rpn-11*	Regulatory particle	BGLB013365-PB	311 aa	NP_494712.1	*Caenorhabditis elegans*	312 aa	9^-172^
*rpn-12*	Regulatory particle	BGLB033779-PA	266 aa	NP_496489.1	*Caenorhabditis elegans*	250 aa	3^-43^
*rpn-13*	Regulatory particle	BGLB002525-PB	315 aa	NP_498387.2	*Caenorhabditis elegans*	374 aa	1^-13^
*rpn-15*	Regulatory particle	BGLB019861-PA	656 aa	NP_609082.1	*Drosophila melanogaster*	908 aa	5^-108^
*rpt-1*	Regulatory particle	BGLB037931-PA	323 aa	NP_506005.1	*Caenorhabditis elegans*	435 aa	1^-146^
*rpt-2*	Regulatory particle	BGLB012308-PB	439 aa	NP_504558.1	*Caenorhabditis elegans*	443 aa	0.0
*rpt-3*	Regulatory particle	BGLB007174-PB	419 aa	NP_498429.1	*Caenorhabditis elegans*	414 aa	0.0
*rpt-4*	Regulatory particle	BGLB007607-PB	392 aa	NP_001022113.1	*Caenorhabditis elegans*	406 aa	0.0
*rpt-5*	Regulatory particle	BGLB005280-PB	428 aa	NP_491672.1	*Caenorhabditis elegans*	430 aa	0.0
*rpt-6*	Regulatory particle	BGLB003566-PC	405 aa	NP_499609.1	*Caenorhabditis elegans*	416 aa	0.0

**Fig. 1:**
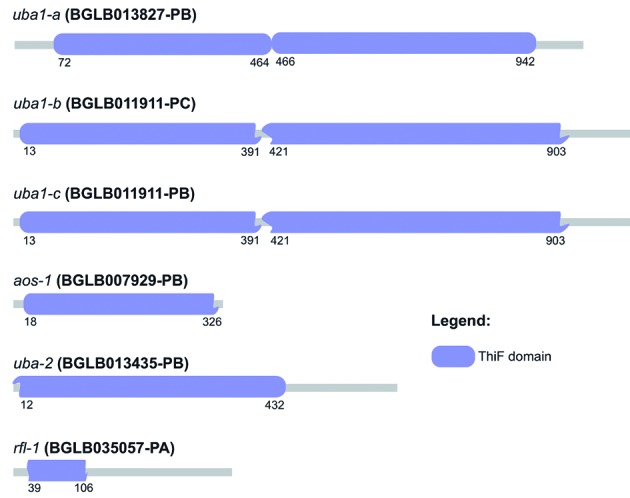
scheme of the presence and distribution of the conserved domain ThiF (CL0063) in E1 sequences identified in the transcriptome of *Biomphalaria glabrata*.

*Ubiquitination pathway* - The analysis performed by PFAM and CDD showed that all the proteins and enzymes found for the ubiquitination pathway in *B. glabrata* have conserved domains and active sites/catalytic clefts/structural motifs. These conserved domains identified for each set of sequences correspond to their previously known structures. In the ubiquitin (BGLB020284-PA) protein, the ubiquitin family domain was identified, as well as in the organisms *D. melanogaster* and *C. elegans* [Supplementary data (Fig. 1A)]. This domain is approximately 72 amino acids in size and shows some important residues for the molecule, such as those involved in the interaction of the molecule with E1 and E2. In addition, lysine residues at specific positions enable the formation of polyubiquitin chains [Supplementary data (Fig. 1B)].

**Fig. 2:**
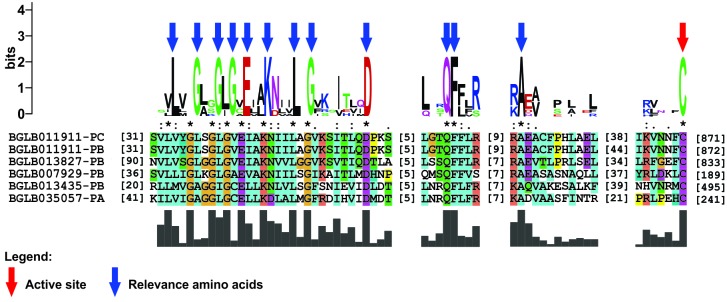
amino acid residues involved in the formation of the active site of the ubiquitin activating enzymes (E1) identified in *Biomphalaria glabrata*. The cysteine residue (C) appears to be the active site (indicated by a red arrow), while the other amino acid residues are likely involved in mediating the catalytic activity (indicated by blue arrows).

In the genes identified as E1, some domains were found distributed along the sequence and were superimposed among them (Table II). These sequences showed the ThiF domain as the main representative showing a fairly characteristic size and location between the sequences of *B. glabrata* (Fig. 1). Additionally, it was possible to observe the presence of a cysteine residue as the active site of these enzymes and other amino acid residues relevant to the performance of the catalytic activity that it possess (Fig. 2). E2 are molecules capable of interacting with the other two classes of enzymes of the ubiquitination pathway because they are in the middle of this cascade. Genes found in the *B. glabrata* encode proteins that shared the UBQ_con domain and have a conserved cysteine residue as the active site, responsible for the role of conjugation of the ubiquitin molecule with the E3 [Supplementary data (Fig. 2A-B)].

**TABLE II:** Distribution of conserved domains found in the E1 sequences of *Biomphalaria glabrata*

Gene	ID sequence	Domain name	Start and end of domain	E-value
*uba1-a*	BGLB013827-PB	ThiF	72-464	3.6^-38^
		E1 FCCH	245-314	4.1^-32^
		E1 4HB	315-383	1.2^-24^
		ThiF	466-942	1.1^-73^
		UBA e1 thiolCys	655-867	2.5^-73^
		E1 UFD	950-1023	5.4^-27^
*uba1-b*	BGLB011911-PC	ThiF	13-391	3.2^-29^
		E1 FCCH	193-263	1.1^-27^
		E1 4HB	265-332	1.3^-16^
		ThiF	421-903	9.8^-64^
		UBA e1 thiolCys	602-854	1.5^-73^
		E1 UFD	925-1010	8.4^-15^
*uba1-c*	BGLB011911-PB	ThiF	13-391	3.2^-29^
		E1 FCCH	193-263	1.1^-27^
		E1 4HB	265-332	1.3^-16^
		ThiF	421-903	9.8^-64^
		UBA e1 thiolCys	602-854	1.5^-73^
		E1 UFD	825-1011	8.1^-17^
*aos-1*	BGLB007929-PB	ThiF	18-326	5.8^-35^
*uba-2*	BGLB013435-PB	ThiF	12-432	1.1^-71^
		UAE Ubl	447-533	1.5^-21^
*rfl-1*	BGLB035057-PA	ThiF	39-106	3.8^-30^
		E2 bind	256-340	5.4^-24^

**Fig. 3:**
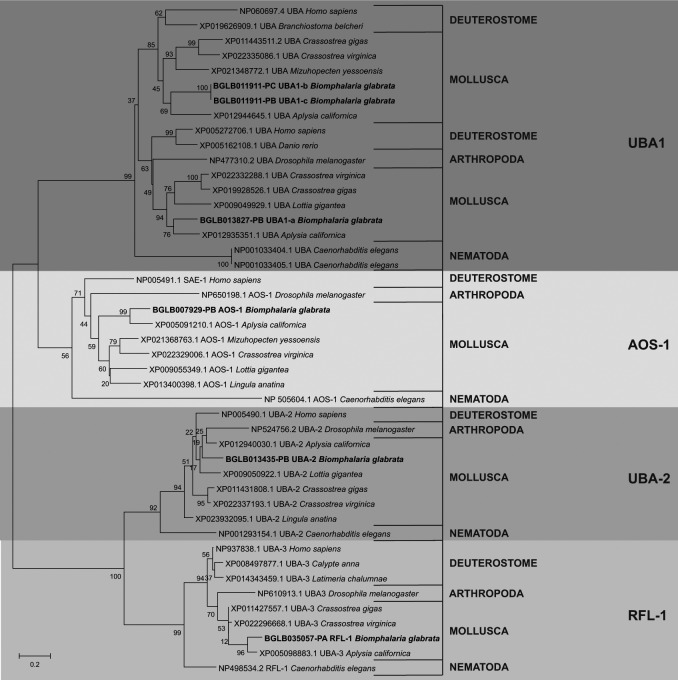
phylogenetic distribution of the E1 sequences found in *Biomphalaria glabrata* data against their orthologous organisms.

**Fig. 4:**
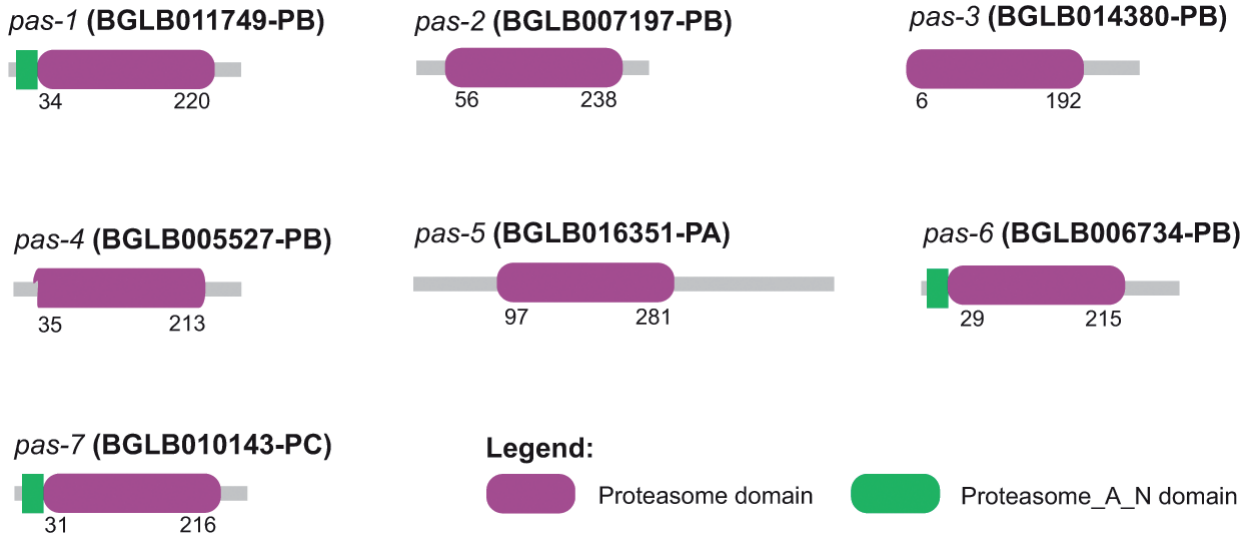
location and distribution of the proteasome domain identified in the sequences of the PAS group of *Biomphalaria glabrata*.

**Fig. 5:**
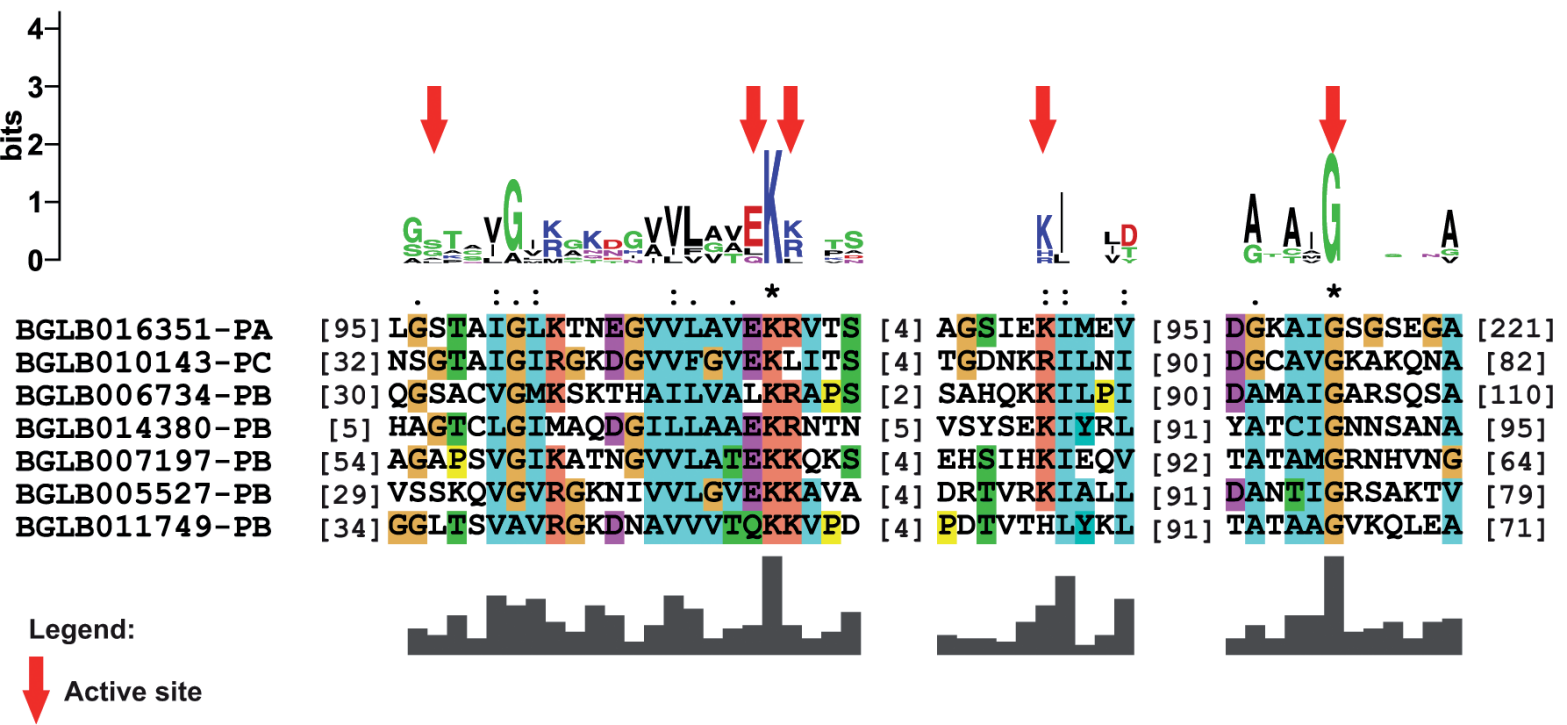
active site found in the sequences participating in the PAS group of the *Biomphalaria glabrata* data. The residues involved in the formation of the site are indicated by red arrows.

The three ubiquitin ligases were divided into HECT, RING finger and U-box and grouped based on conserved domains HECT domain, U-box and domains homologous to RING finger, demonstrating the conservation of the catalytic activity of each group of enzymes [Supplementary data (Figs 3A, 4A, 5A)]. The analyses performed by the CDD show that the proteins of the HECT group had a catalytic cleft formed from the presence and distribution of some amino acid residues in specific and conserved positions [Supplementary data (Fig. 3B)]. The proteins classified in the RING finger and U-box groups evidenced the formation of a structural motif for each group. For the RING finger protein group, the cysteine residue was conserved in all *B. glabrata* genes [Supplementary data (Fig. 4B)]. On the other hand, the motif found in the U-box gene group was determined by the distribution of various residues located along the sequences, but there was a highly conserved aspartic acid residue [Supplementary data (Fig. 5B)].

**Fig. 6:**
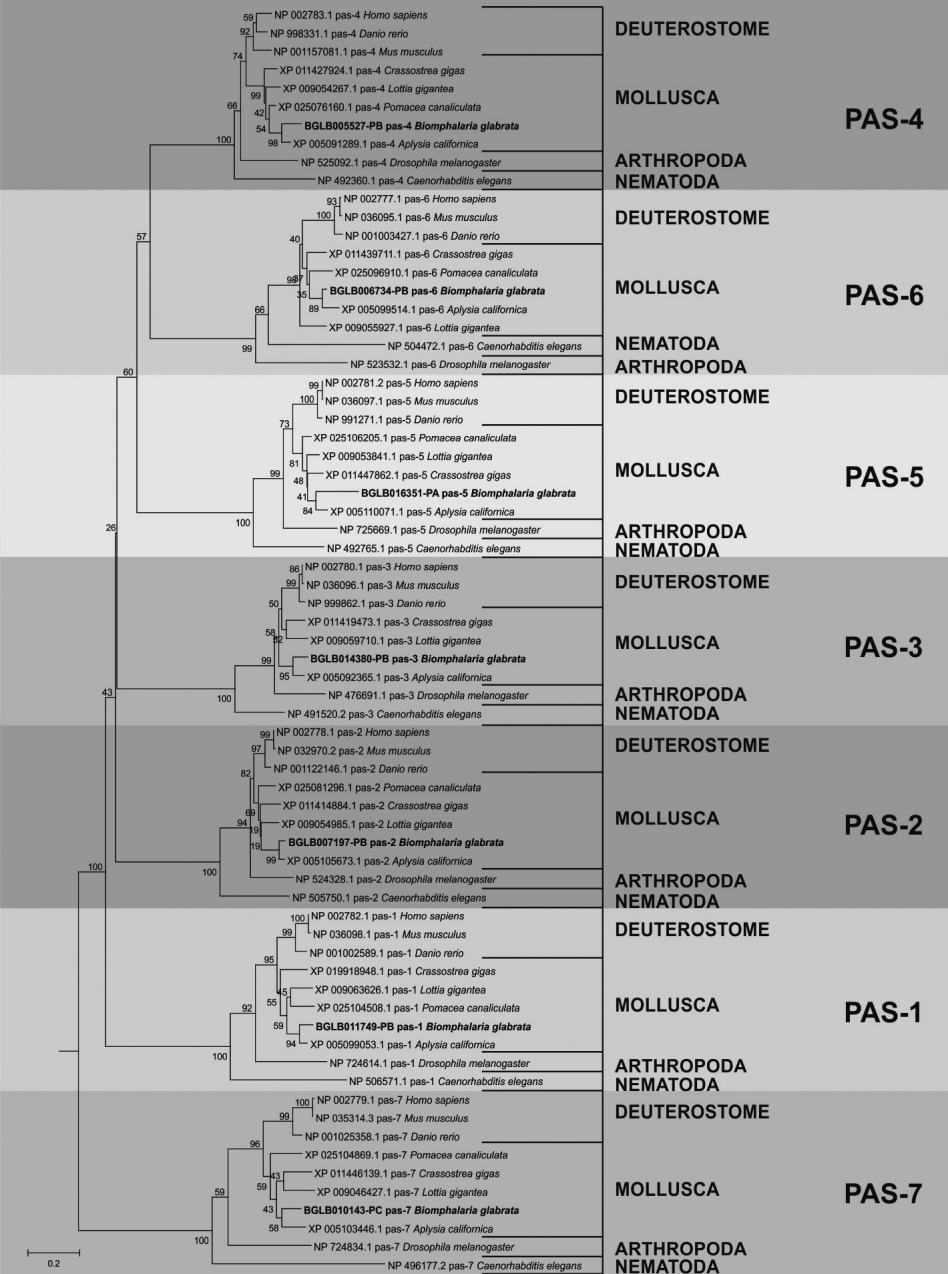
phylogenetic tree generated by the analyses using the sequences identified as PAS of mollusks versus model organisms and their orthologs.

**Fig. 7:**
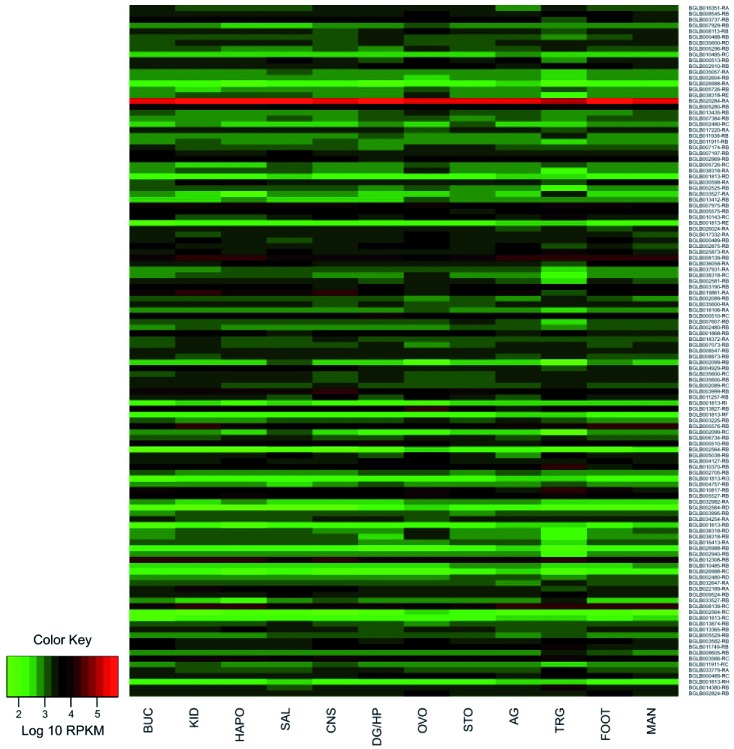
expression profile of the genes involved in UPS in 12 different tissues of the adult snail.

Phylogenetic analysis was performed to evaluate the evolutionary relationship of the genes found in *B. glabrata* against the genes of orthologous organisms and to demonstrate the degree of conservation. For all classes of enzymes, this result showed that there was a very organised division between the clades of the enzymes and that they were distributed evolutionarily as presented by the evolutionary tree of life [Supplementary data (Figs 6, 7, 8, 9)]. This infers that the sequences found in the snail were likely enzymes involved in the ubiquitination pathway. In addition, the distribution found among the organisms used for this analysis was demonstrated in Fig. 3, showing the analysis done for E1.

*26S proteasome formation* - The genes involved in proteasome formation identified in *B. glabrata* were divided between regulatory particles and core particles. Groups called PAS and PBS were related to the nucleus of the molecule, while the RPT and RPN groups form the recognition portions. All proteins encoded from the genes of the PAS showed the proteasome conserved domain (Fig. 4) and an active site consisting of amino acid residues distributed at five specific positions throughout the seven sequences (Fig. 5). In the same sense, the PBS group was also shown to share the proteasome conserved domain in their sequences and, in addition, showed the presence of an active site consisting of amino acid residues in seven distinct positions, but with low conservation [Supplementary data (Fig. 10A-B)]. This feature may indicate a direct site-position relationship and not between the site and amino acid conservation.

The other identified proteins were divided into the RPT and RPN groups that formed the regulatory and recognition portion of 26S proteasome. The protein group called RPT showed no amino acid residues relevant to active site formation; however, all six sequences presented the conserved domain AAA in the C-terminal portion [Supplementary data (Fig. 11)]. Unlike the RPT group, the sequences of the RPN group did not share the common domain [Supplementary data (Fig. 12)] and therefore did not show a conserved active site.

The phylogenetic analysis performed for the sequences involved with the formation of 26S proteasome showed an evolutionary distribution that corroborates with that known through the tree of life of the animals. In addition the genes identified in *B. glabrata* and their orthologous from other organisms were organised in deuterostomes and protostomes as seen for the results found in the phylogeny concerning the ubiquitination pathway [Supplementary data (Figs 13, 14, 15)]. Fig. 6 shows the phylogenetic distribution of the PAS group using those identified in the snail and the orthologous genes.

*Expression profile of the genes involved in the UPS identified in B. glabrata* - The heatmap demonstrated the expression profile of all 119 sequences found in the *B. glabrata* in 12 libraries of tissues: albumen gland (AG), buccal mass (BUC), central nervous system (CNS), digestive gland/hepatopancreas (DG/HP), muscular part of the headfoot (FOOT), heart including amebocyte producing organ (HAPO), kidney (KID), mantle edge (MAN), ovotestis (OVO), salivary gland (SAL), stomach (STO) and terminal genitalia (TRG). The vast majority of the UPS related genes presented low to medium expression for all libraries used (Fig. 7).

The ubiquitin (*ubq-1*) gene, identified as BGLB020284-RA was the only one presented highly expressed in all libraries. Other genes such as *rpt-2* (BGLB012308-RB), *wwp-1-a* (BGLB008139-RC) and *wwp-1-b* (BGLB008139-RB) also demonstrated sufficient, but not high, expression in all libraries. However, most other genes were not able to show a strong expression profile for any of the libraries. Only a few transcripts such as *pbs-3* (BGLB010370-RB), *uba1-a* (BGLB013827-RB) and *rpn-10* (BGLB003999-RB) were able to display good expression in specific libraries such as TRG, OVO and CNS, respectively (Fig. 7).

The genes with lower expression indicated in the results of the heatmap were related to the class of ubiquitin ligases (E3). All isoforms of the *chn-1* genes (BGLB001813-PI, BGLB001813-PH, BGLB001813-PG, BGLB001813-PF, BGLB001813-PE, BGLB001813-PD, BGLB001813-PC and BGLB001813-PB) and *traf-6* (BGLB026988-PC, BGLB026988-PB and BGLB026988-PA) demonstrated a very low RPKM-based expression profile. In the same sense, a very low expression was perceived for the isoforms of the *fizzy* genes (BGLB002564-PD, BGLB002564-PC, BGLB002564-PB, BGLB005726-PB and BGLB005726-PC) and *skpt-1* (BGLB032982-PA) that were involved in the formation of E3 complexes as target recognising subunit (Fig. 7).

## DISCUSSION

The ubiquitin-proteasome system (UPS) is one of the most conserved components among eukaryotic organisms and has been described in model organisms such as *D. melanogaster*, *C. elegans* and *Homo sapiens*. Most sequences identified in *B. glabrata* have an amount of amino acids close to or even equal to the number of amino acids found in the proteins of the model organism used, *C. elegans* and/or *D. melanogaster.* Ubiquitin is a polypeptide with 76 amino acids in its structure capable of labeling target substrates.^21^ However, the gene identified in *B. glabrata* encodes a gene product of 229 amino acids due to a polygene responsible for translating this molecule, a fusion of the ubiquitin peptides. In addition, the same situation in *C. elegans* and *D. melanogaster* occurs [Supplementary data (Fig. 1A)]. The difference found in the size of these sequences can be explained by the annotation performed for the mollusk sequence, suggesting that a new annotation process should be performed. This situation refers to the existence of polygenes that have tandem repeats of the coding region of ubiquitin in their sequences. Thus, most organisms present sequences of polygenes for ubiquitin and from that, they have become one of the best models for the study of the evolution of gene clusters.^21^ All three organisms used for this analysis have the conserved ubiquitin family domain (PF00240) in the same position within the polypeptide sequence, showing that the presence and location of the domain in the sequence may be of great importance for the role that this polypeptide plays.

The ubiquitin family domain in *B. glabrata* proteins shares very important and highly conserved amino acids against the orthologous organisms. Some of these residues have the ability to form a site of interaction with the ubiquitin conjugating enzymes (E2) and to catalyse the transfer [Supplementary data (Fig. 1B)]. Another three lysine (K) residues play a crucial role in labeling the target substrate. These residues at positions 29, 48 and 63 of the domain are considered binding sites for novel ubiquitin molecules capable of forming polyubiquitin chains. The chains formed at positions 29 and 48 induce degradation by the proteasome, while the chains originating at the lysine residue at position 63 are, for instance, related to DNA damage response signaling.^22^

It was not possible to trace the evolutionary history of ubiquitin through phylogenetic analysis because it is an extremely conserved sequence [Supplementary data (Fig. 1B)]. There is a high degree of conservation involving the ubiquitin encoding gene, suggesting that it is able to undergo few combined evolutionary events. The reason why this conservation is so high is still not fully understood, but the properties presented by the ubiquitin molecule in the cells were selected and fixed in a eukaryotic ancestor in the early stages of evolution.^23^

E1 are the first enzymes recruited to the ubiquitination pathway and play the role of activating the ubiquitin molecule to be transferred to the substrate. Activation occurs by the ubiquitin adenylation from an ATP-dependent thioester bond between the E1 cysteine residue and the glycine of the C-terminal portion of ubiquitin. The activated ubiquitin molecule is transferred to an E2.^7^ ^,^ ^8^ ^,^ ^10^ An existing hypothesis is that the E1 found in eukaryotes have evolved from the bacterial enzymes MoeB and ThiF.^24^ All sequences identified as E1 in *B. glabrata* showed the ThiF domain with a similar amount of amino acids, showing a high degree of conservation, except in the sequence translated from the *rfl-1* gene, which may have incomplete annotation. However, other conserved domains were also found in these sequences (Table II), capable of assisting in the process of ubiquitin activation. A highly conserved cysteine (C) residue was observed as an active site among all sequences identified as E1 in *B. glabrata*, the acceptor portion of ubiquitin in the enzyme. In addition, other amino acid residues in close positions are capable of aiding in the formation of the catalytic site and consequently in the activity that the enzyme plays (Fig. 2). Thus, these residues are probably involved with ATP binding and the formation of the thioester intermediate, activating the ubiquitin molecule for a subsequent transfer to E2.^7^ ^,^ ^8^ ^,^ ^10^ ^,^ ^23^

E2 are enzymes from the centre of the ubiquitination pathway and are related to the other two enzymatic classes in the cascade. In addition, they play the role of catalysing the binding reaction of the ubiquitin molecule with the target substrate.^23^ The UQ_con domain was identified in all sequences identified as E2 present in the *B. glabrata* data, sharing close and highly conserved sizes. On the other hand, the UBA domain was found only in the sequence encoded from the *ubc-20* gene (BGLB036058-PA) and has a role in limiting the formation of the polyubiquitins chain [Supplementary data (Fig. 2A)]. In addition, these enzymes have a cysteine residue as active site capable of binding the ubiquitin molecule to E2 and mediates the interaction with E3.^23^ Other amino acid residues were important in the sequences because they were related to the interaction E2 makes with E3; however, they did not show a high degree of conservation [Supplementary data (Fig. 2B)].

E3 performs the process of transferring the ubiquitin to the N-terminal portion of the lysine residue (K) belonging to the target substrate, and is more related to the target substrate it recognises than to the ubiquitin molecule. E3 is encoded in hundreds of proteins in eukaryotic cells, allowing the labeling of different proteins in specific ways; they are organised into different classes (Table I).^25^ Thus, each of these classes shares specific domains that differ by recognition of the E2-ubiquitin complex. All E3-HECT sequences identified in *B. glabrata* showed a fairly conserved HECT domain in their C-terminal portion, maintaining a position pattern at the end of the protein chain. In the HECT domain, it was possible to find amino acid residues involved in the formation of a catalytic cleft and a cysteine as active site [Supplementary data (Fig. 3B)] where a thiol linkage between ubiquitin and ligase is performed. Although this residue was present in the C-terminal portion of the domain, its N-terminal portion is responsible for interacting with E2 and with the substrate that is bound in distinct regions along the enzyme sequence and outside the domain region.^26^ However this binding does not happen directly, because a recruitment of the E2-ubiquitin complex is first made to the active site of the ligase. Subsequently, ubiquitin is transferred to the lysine residue belonging to the target substrate through a transesterification reaction.^22^ ^,^ ^23^ ^,^ ^26^

The E3-RING finger class has a direct transfer of ubiquitin, since concomitant recruitment between the E2-ubiquitin complex and target substrate is performed. This mediates and facilitates the formation of the binding between ubiquitin and the target protein.^22^ ^,^ ^26^ ^,^ ^27^ The conserved domains zf-C3HC4_3 (PF13920), zf-MIZ (PF02891), zf-RING_2 (PF13639), Sina (PF03145), Prok-RING_4 (PF13923) and zf-C3HC4_2 (PF14447) are homologous to the RING finger domain [Supplementary data (Fig. 4A)] and were found distributed among all the sequences identified in the mollusk. A highly conserved distribution of cysteine, cysteine, histidine and cysteine was found in the results of this work, providing evidence that there was a structural motif formation [Supplementary data (Fig. 4B)] and that it is related to the bond made with zinc.^28^

Like the E3-RING finger, the E3-U-box class also recruits the E2-ubiquitin complex and target substrate concomitantly. In addition, they share a common organisation and architecture, suggesting that U-box domains are modified RING domains because they do not bind to zinc and are formed from hydrogen bonds.^29^ Thirteen of the fifteen E3-U-box sequences identified in *B. glabrata* demonstrated the U-box domain (CL0229) and the other two inferred other correlated domains [Supplementary data (Fig. 5A)]. The *ufd-2* (BGLB026024-PA) sequence showed only the Ufd2P_core domain (PF10408) which escapes ubiquitinated proteins to the proteasome and was directly linked to the U-box domain in the C-terminal portion of the sequence. The other sequence was designated *cyn-4* (BGLB022189-PA), which was found the Rtf2 domain (PF04641), similar to the RING finger domain, but has a three-dimensional ring-like structure and only one site for binding with zinc. The absence of U-box domain may be related to the annotation process of these sequences. The results obtained showed that in the U-box domain there exists the formation of a structural motif in which not all amino acid residues were highly conserved, but there are some that were present in all sequences used in the analysis [Supplementary data (Fig. 5B)].

The proteasome is a proteolytic complex formed by an association between a 19S regulatory particle (PA700) and a central component 20S, composed of approximately 70 subunits. The 19S particle is divided into base and lid and are reversibly bound to 20S. The regulatory portion recognises the labeled substrate while the 20S nucleus is responsible for degrading the protein in minor peptides.^7^ ^,^ ^14^

Nineteen genes from the RPN and RPT groups were identified in our analysis (Table I), encompassing sequences of major importance such as RPN-11, RPN-15 and RPN-3, involved with target substrate recognition and removal of the ubiquitin chain formed.^15^ ^,^ ^30^ In the same sense, some RPT subunits, such as *rpt-2* and *rpt-5* were also found in the data of *B. glabrata*; however, they are related to activation of the 20S nucleus.^31^ The results of this work show that RPT shares the conserved AAA domain, but not of an active site, structural motif or catalytic cleft, inferring that the function of these molecules was linked to the sequence that the conserved domain possesses [Supplementary data (Fig. 11)]. Conversely, sequences from the RPN group did not have a common domain and did not present any conserved active sites [Supplementary data (Fig. 12)]. A suggested explanation for this feature was that these subunits may be more involved with the target substrate than with the catalytic portion of the 26S proteasome and therefore do not share highly conserved sequences.

The nucleus of the proteasome is composed of α and β subunits. Subunits of the α-type have a structural and regulatory role, whereas β possesses proteolytic and degradation activity. Fourteen sequences involved in the formation of the 20S proteasome between α and β subunits were identified in *C. elegans*,^31^ corroborating the number of sequences found in *B. glabrata* data (Table I). All sequences belonging to the PAS (α-subunit) and PBS (β-subunit) groups have the conserved proteasome domain in their N-terminal portions [Fig. 4 and Supplementary data (Fig. 10A)], demonstrating that the presence of this domain in this region is important for the activity they play. An active site composed of five relatively conserved amino acid residues was identified in the results obtained for the PAS group genes among all sequences (Fig. 5). Conversely, for the PBS group, a less conserved active site was found, which may be involved in the proteolytic function of this subunit [Supplementary data (Fig. 10B)].

Through the phylogenetic analysis, it was possible to describe the evolutionary history of the sequences under study and from these results to infer their identity by the location in the generated tree, as well as the proximity to orthologous organisms and model organisms. Our results show a clear and specific distribution among all data obtained from the analysis performed using MEGA5.2. The evolutionary organisation of each set of sequences, both the sequences involved in the ubiquitination pathway and those related to the formation of the 26S proteasome (Figs 3, 6), corresponds to what was already known in the animal tree of life. There was a well-defined division between the clade of deuterostomia and protostomia organisms, separated between phyla, and grouped according to the location of the species in the evolutionary process.^32^ In the same sense, each sequence identified in *B. glabrata* presents well-defined ramifications involving its orthologous organisms, providing strong evidence that the putative sequences found in the mollusk transcriptome are real sequences involved in the ubiquitin-proteasome complex.

Our results demonstrate that the gene responsible for translating the ubiquitin (*ubq-1*) molecule was the only one to present a high expression profile in the RNAseq data of all 12 libraries used, that is, in all analysed tissues of the adult snail. Ubiquitin is a key molecule in UPS; however, it is also involved in other cellular signaling processes such as plasma membrane transport and DNA repair.^22^ This only happens because of the role it plays in labeling target substrates with one or more ubiquitins, forming a polyubiquitin chain. In addition to these functions, ubiquitin is also involved in autophagy.^18^ ^,^ ^22^

The high expression that the *ubq-1* transcript has demonstrated can be explained by the diversity of cellular processes in which ubiquitin is involved, being related to virtually all cellular processes, directly or indirectly. This also explains the high expression in all organs of *B. glabrata* used for analysis. In addition, the formation of polyubiquitin chains influences that a greater amount of this gene was transcribed and may be translated, since more than one molecule of ubiquitin is required to label only a target substrate.

Most of the genes involved in UPS and identified in the *B. glabrata* genome presented a median expression for all organs of uninfected adult snails evaluated. This expression profile infers that both the transcripts involved in the ubiquitination pathway and genes participating in the formation of the 26S proteasome do not show a high expression under normal conditions of mollusk survival. Thus, it can be inferred that these transcripts share behavior related to the expression very similar for all analysed organs. Our results indicate that this is a pathway capable of performing its role in the organism without large amounts of circulating molecules in its adult state. Our results were based on uninfected snails, although the hypothesis is supported that the results obtained for these transcripts may be different in infected snails, demonstrating degrees of expression different from those presented in this work, since the UPS plays a role related to immunity. To confirm this hypothesis, other transcriptomic analyses are required. Some transcripts appear to have a direct relationship with specific tissues because they have a very low expression profile for 11 libraries and relatively high for only one, suggesting that the function they play may be linked to their location in the organism. For example, *rpn-10* (BGLB003999-RB) demonstrates better expression in the central nervous system (CNS) than in the other tissues (Fig. 7). The deletion of *rpn-10* in *D. melanogaster* leads to lethality and demonstrates cause abnormalities in the mitotic cycle such as aneuploidies and absence of chromosomal segregation in CNS larval cells, besides accumulating multiubiquitin proteins in the tissue, since this is a gene participant of the 26S proteasome regulatory portion.^33^ In view of this, it would be interesting to investigate in depth the relationship between the transcripts identified in *B. glabrata* and the snail tissues demonstrated in this work. This is due to the fact that they are tissue-specific transcripts in which their functions are performed more efficiently in exclusive tissues, demonstrating their importance in the development of these organisms.

Four families of genes showed a low expression for all libraries used. All were involved with ubiquitin ligases (E3), two of which refer to transcripts of E3 enzymes and two were genes encoding molecules that form part of complexes. The genes encoding the ubiquitin ligase enzymes are transcribed from the genome in the hundreds. This means that many different E3 are expressed, but not highly expressed, since they are directly related to recognition of the target substrate.^7^ ^,^ ^10^ In the same sense, because of the specificity they present for the labeled substrate, they do not need to be transcribed in large quantities into the mollusk tissues in non-stress situations. *traf-6* is in a family of genes that presented one of the lowest expression levels according to the results; however, others studies have related this transcript to cellular signaling in the immune system.^34^ Thus, the low expression can be explained by the fact that the data from the analysed libraries refer to uninfected adult snail transcripts. *traf-6* becomes a possible gene to be investigated in future research using data from mollusks under infection conditions, suggesting that this expression may be increased in front of the parasite. In addition to *traf-6*, other genes involved in the ubiquitination pathway found in our results have also been described involved with immunity, such as *diap1* (BGLB013412-RB) and *ubc-13* (BGLB033527-RA and BGLB033527-RB). All presented a moderate expression profile, which suggests that genes may have a differential expression in case of infection.

Our results allow the beginning of the search for new knowledge and perspectives involving *B. glabrata*, increasing the amount of information on the regulation of the mollusk and the relation between parasite/host. This work provides evidence of the presence of the UPS in the genome and transcriptome of the snail, besides offering a delineation of the expression profile of the 119 sequences identified for mollusks in their normal development. Thus, it is concluded that the UPS is a system conserved in *B. glabrata* and that ubiquitin is indeed the key molecule of the system, demonstrating a high expression for all tissues analysed in adult snails. In the normal state of survival, the UPS is a moderately expressed system as a whole, but is believed to have a different expression in infected organs. To obtain these results, new transcriptomic analyses are required. Our work also provides a basis for new hypotheses to be developed, such as the evaluation of the expression profile of these sequences in different phases of the life of the organism, as well as new studies involving the analysis of the behavior of this pathway when the intermediate host is infected by *S. mansoni*. Therefore, these results may offer new ways of controlling infection and, consequently, schistosomiasis.
