# Supplementary material for: Genome-wide identification, characterisation and expression profiling of the ubiquitin-proteasome genes in Biomphalaria glabrata
Source: Mem Inst Oswaldo Cruz. 2019 Jun 3;114:e190052. doi: 10.1590/0074-02760190052 (PMC6548493; doi:10.1590/0074-02760190052)
Supplement: Supplementary file 1 [file 1678-8060-mioc-114-e190052-s.pdf]

TABLE

RNAseq data libraries of 12 adult snail tissues used in expression analyses for heatmap map generation

RNAseq Ilumina sequence datasets						
Accession ID	BioSample ID	Library name	Read type	Sequence platform	Library type	Insert/Read length
SRX648260	SAMN02905163	BGAC-100819_OVO_TotalRNA_Tube3	Illumina	HiSeq 2000	RNAseq Paired-end	NA/100bp
SRX648261	SAMN02905164	BGAC-100819_DG_HP_TotalRNA_Tube6	Illumina	HiSeq 2000	RNAseq Paired-end	NA/100bp
SRX648262	SAMN02905165	BGAC-100819_STO_TotalRNA_Tube8	Illumina	HiSeq 2000	RNAseq Paired-end	NA/100bp
SRX648263	SAMN02905166	BGAC-100819_AG_TotalRNA_Tube11	Illumina	HiSeq 2000	RNAseq Paired-end	NA/100bp
SRX648264	SAMN02905167	BGAC-100819_BUC_TotalRNA_Tube37	Illumina	HiSeq 2000	RNAseq Paired-end	NA/100bp
SRX648265	SAMN02905168	BGAC-100819_KID_TotalRNA_Tube24	Illumina	HiSeq 2000	RNAseq Paired-end	NA/100bp
SRX648266	SAMN02905169	BGAC-100819_HAPO_APO_TotalRNA_Tube15	Illumina	HiSeq 2000	RNAseq Paired-end	NA/100bp
SRX648267	SAMN02905170	BGAC-100819_SAL_TotalRNA_Tube32	Illumina	HiSeq 2000	RNAseq Paired-end	NA/100bp
SRX648268	SAMN02905171	BGAC-100819_CNS_TotalRNA_Tube35	Illumina	HiSeq 2000	RNAseq Paired-end	NA/100bp
SRX648269	SAMN02905172	BGAC-100819_TRG_TotalRNA_Tube16	Illumina	HiSeq 2000	RNAseq Paired-end	NA/100bp
SRX648270	SAMN02905173	BGAC-100819_MAN_TotalRNA_Tube22	Illumina	HiSeq 2000	RNAseq Paired-end	NA/100bp
SRX648271	SAMN02905174	BGAC-100819_FOOT_TotalRNA_Tube28	Illumina	HiSeq 2000	RNAseq Paired-end	NA/100bp

Total RNA was extracted from 12 different tissues/organs dissected from several individual adult BB02 *Biomphalaria glabrata* snails (between 2 and 10 snails per sample to obtain sufficient amounts of RNA). Illumina RNAseq was used to generate tissue-specific transcriptomes for albumen gland (AG); buccal mass (BUC); central nervous system (CNS); digestive gland/hepatopancreas (DG/HP); muscular part of the headfoot (FOOT); heart including amebocyte producing organ (HAPO); kidney (KID); mantle edge (MAN); ovotestis (OVO); salivary gland (SAL); stomach (STO); terminal genitalia (TRG).

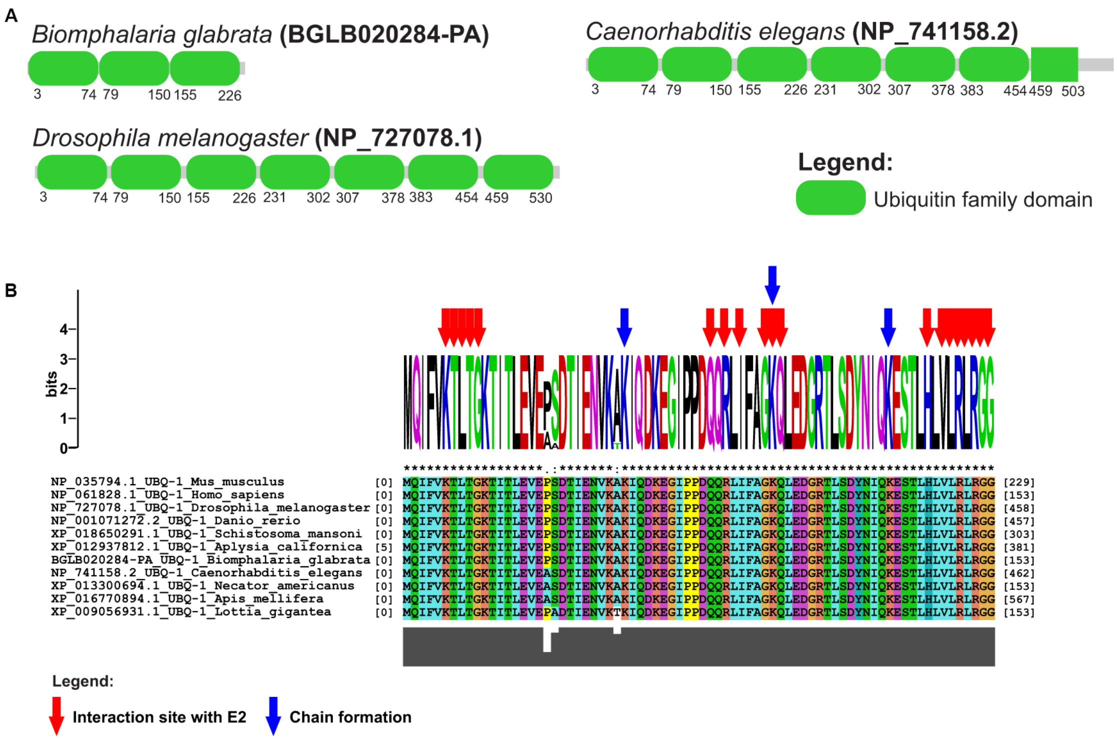

Fig. 1: (A) position of the conserved ubiquitin family domain in the ubiquitin sequences of *Biomphalaria glabrata*, *Caenorhabditis elegans* and *Drosophila melanogaster*. (B) Amino acid residues involved in the formation of the active site and ubiquitin chain.

cc BY

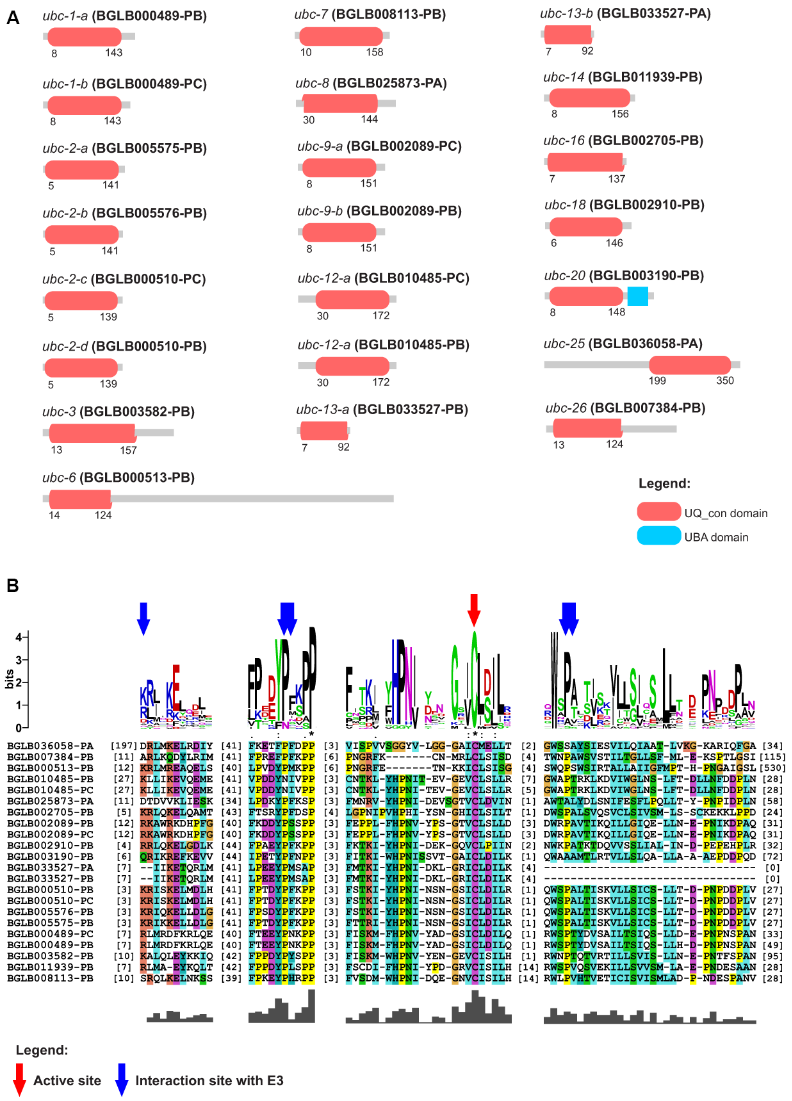

Fig. 2: (A) distribution of the conserved domain UQ_con in the sequences of *Biomphalaria glabrata* identified as E2. (B) Formation of the active site and the interaction with E3 shown by arrows on the amino acid residues.

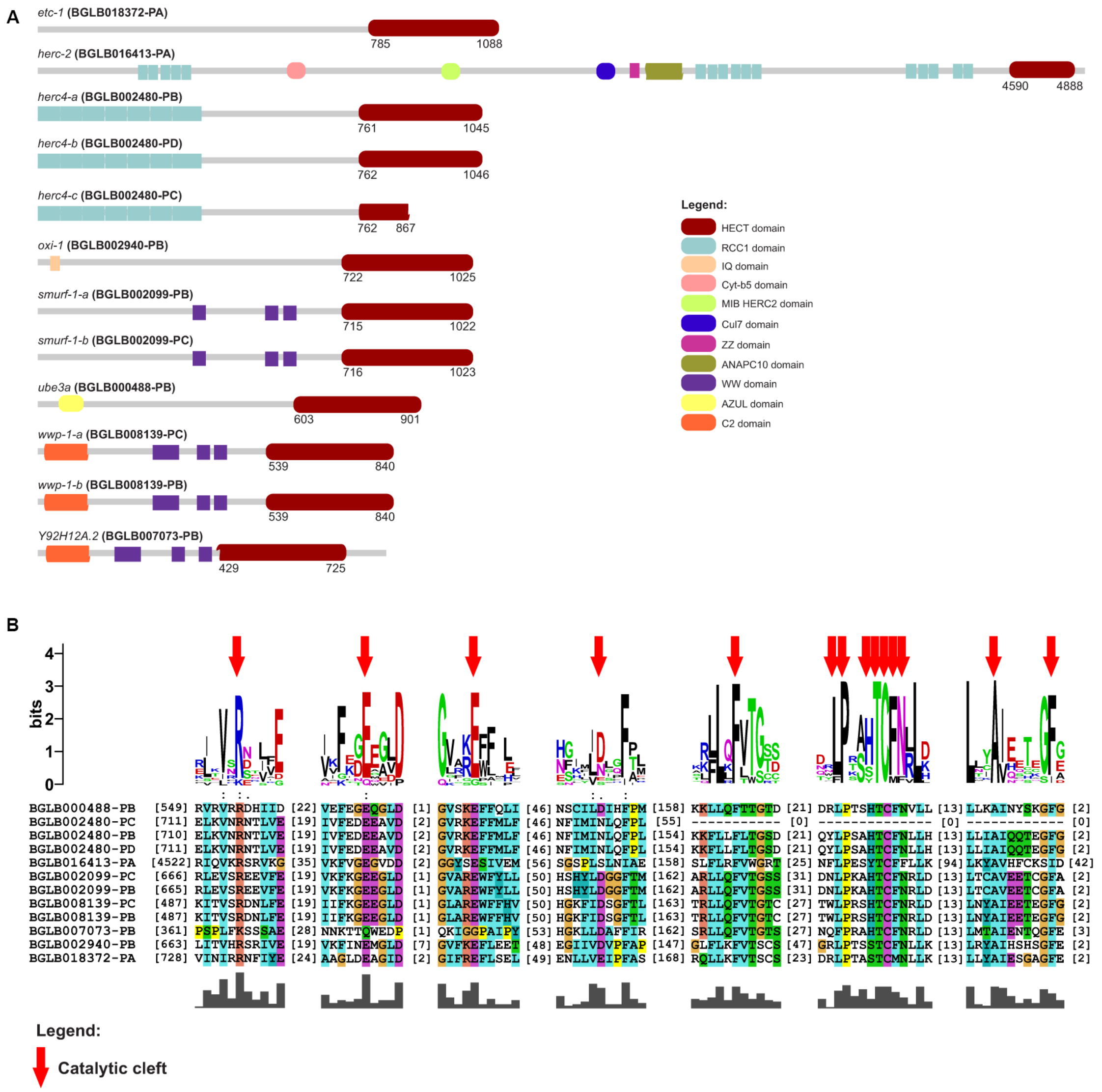

Fig. 3: (A) presence and distribution of the domains conserved found in the sequences of *Biomphalaria glabrata* identified as E3-HECT. (B) Catalytic cleft indicated by arrows on the amino acid residues involved in its formation.

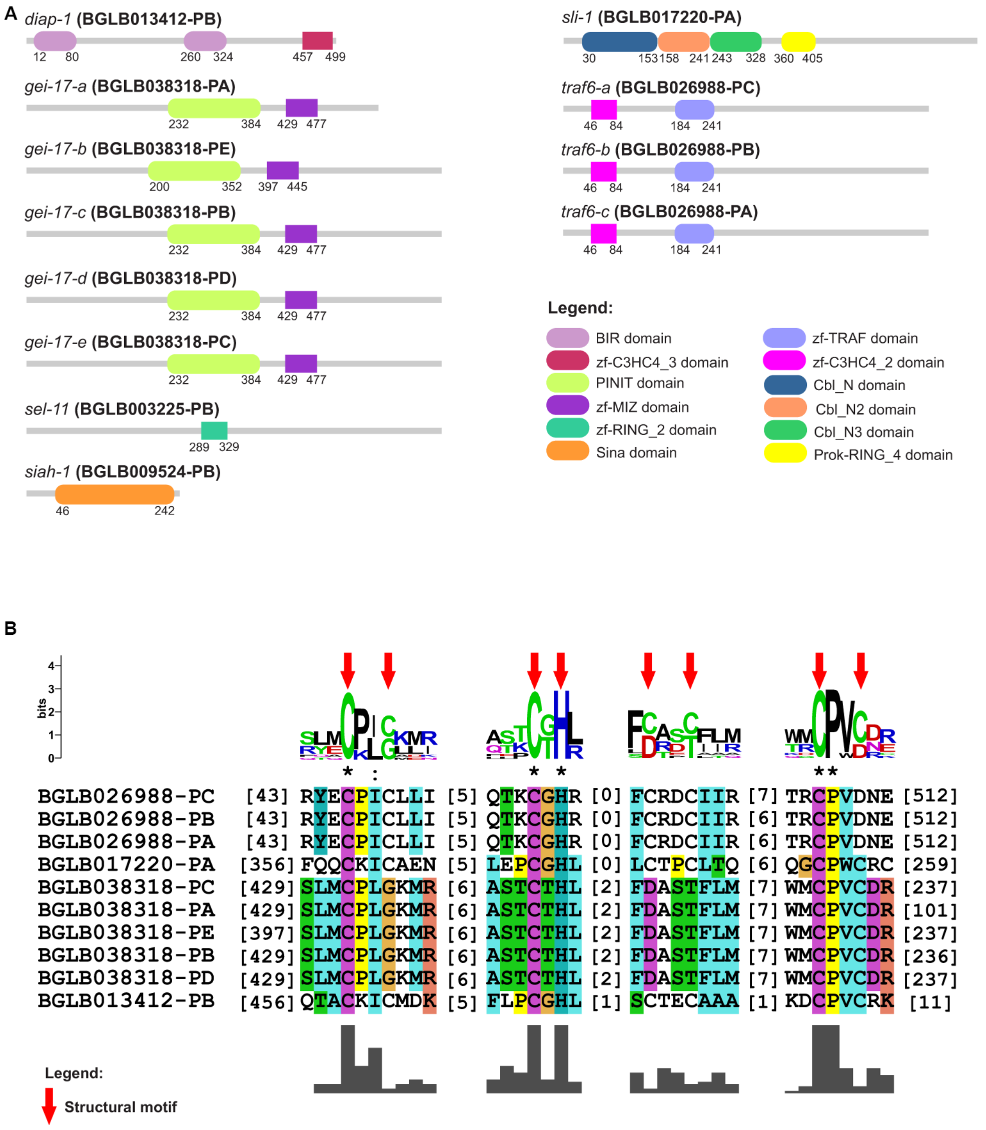

Fig. 4: (A) domains homologous to RING fingers represented in their positions and distributions in the E3-RING finger sequences of *Biomphalaria glabrata*. (B) Amino acid residues and their positions related to the formation of the structural motif found in these sequences.

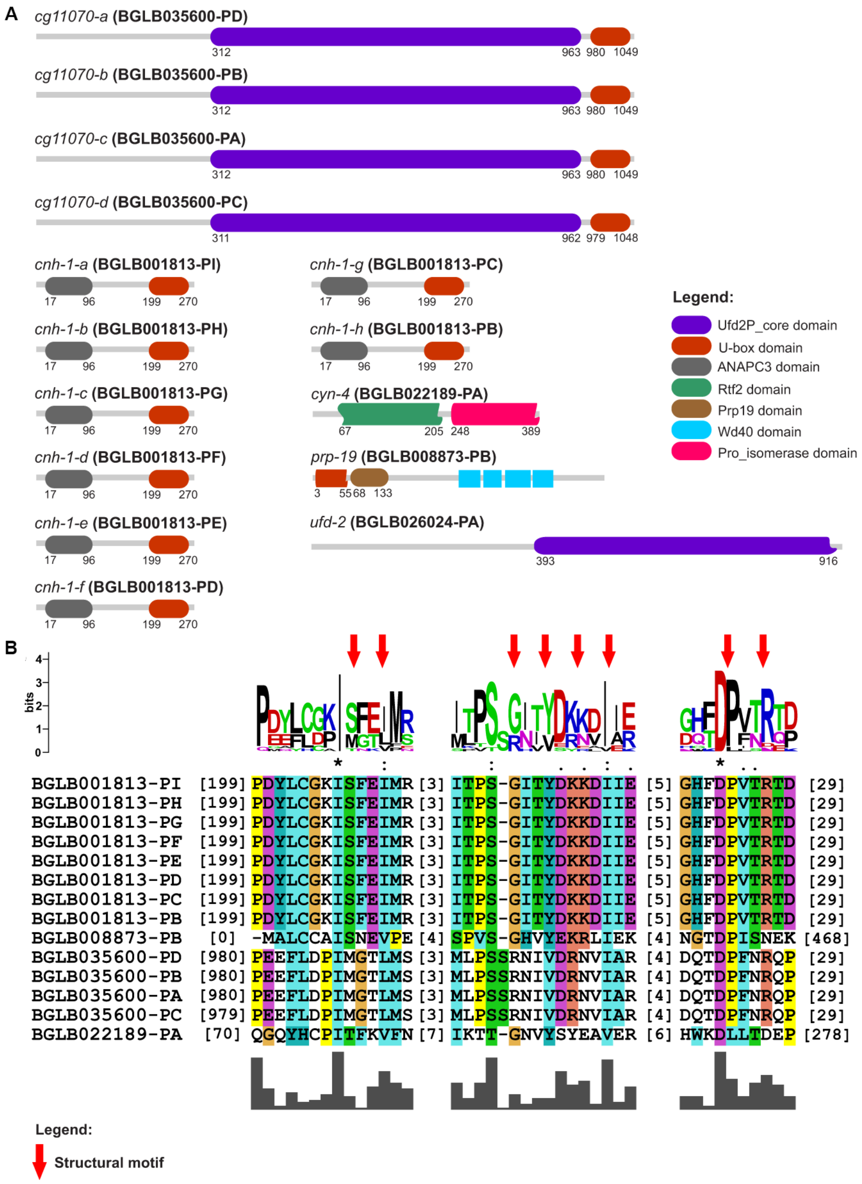

Fig. 5: (A) demonstration of the presence and distribution of conserved domains present in *Biomphalaria glabrata* E3-U-box sequences. (B) Amino acid residues and their positions related to the formation of the structural motif.

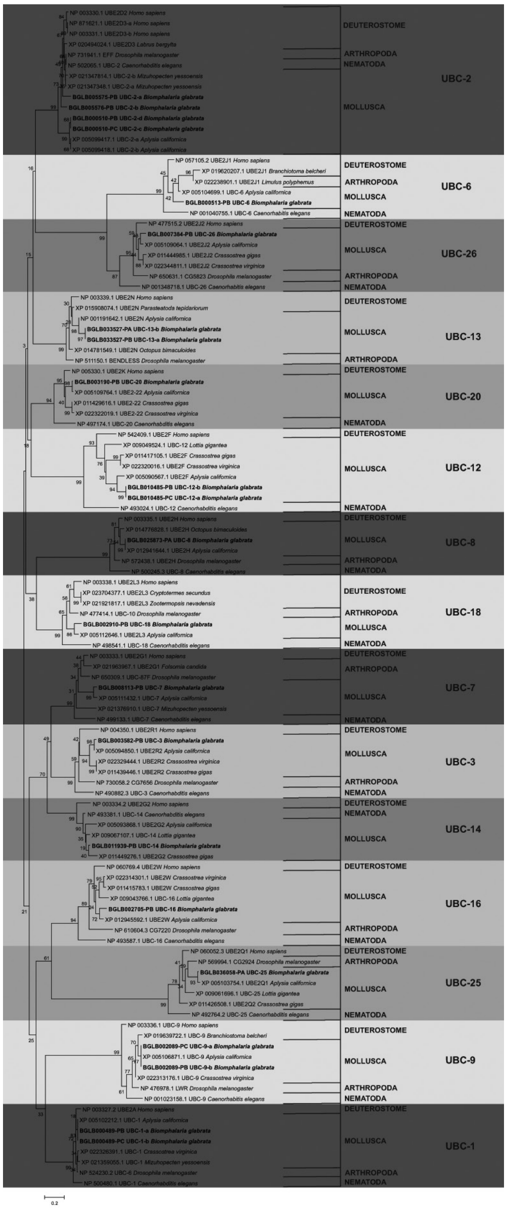

Fig. 6: phylogenetic tree generated from the E2 sequences identified in *Biomphalaria glabrata* and its orthologs.

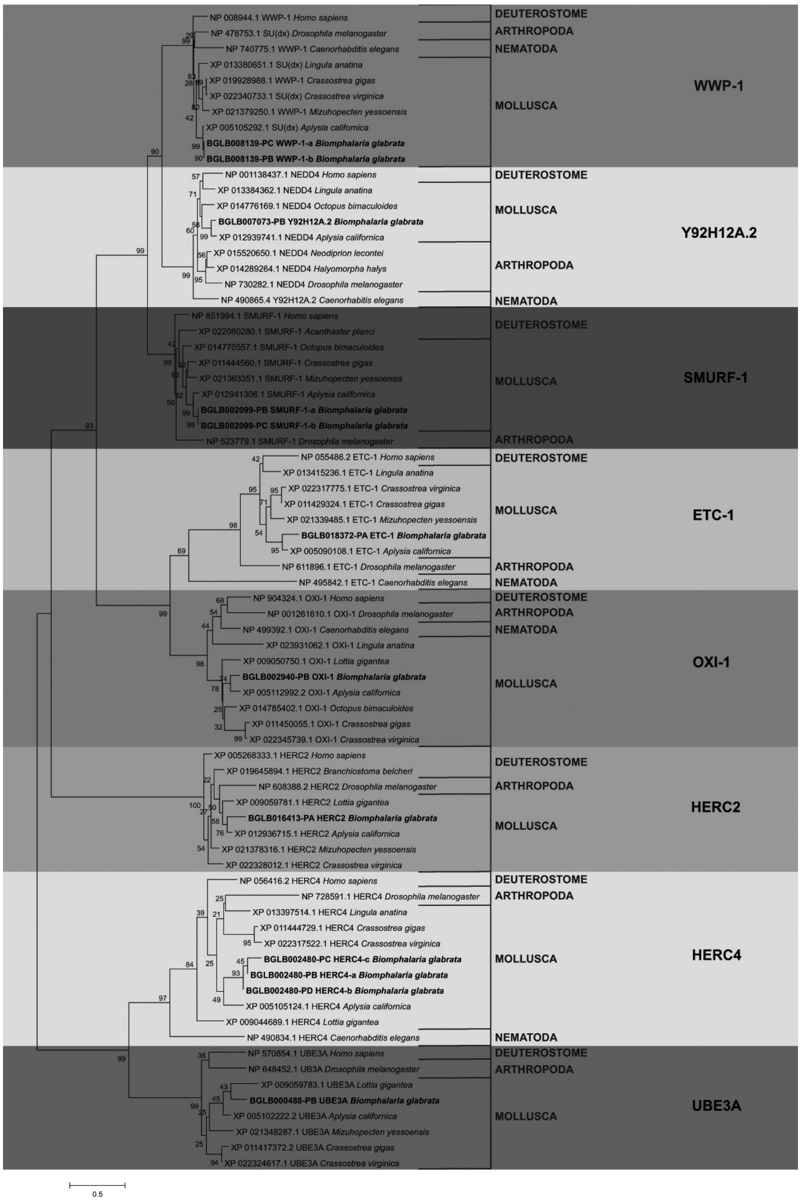

Fig. 7: representation of the evolutionary distribution of E3-HECT sequences for *Biomphalaria glabrata* against their orthologous organisms.

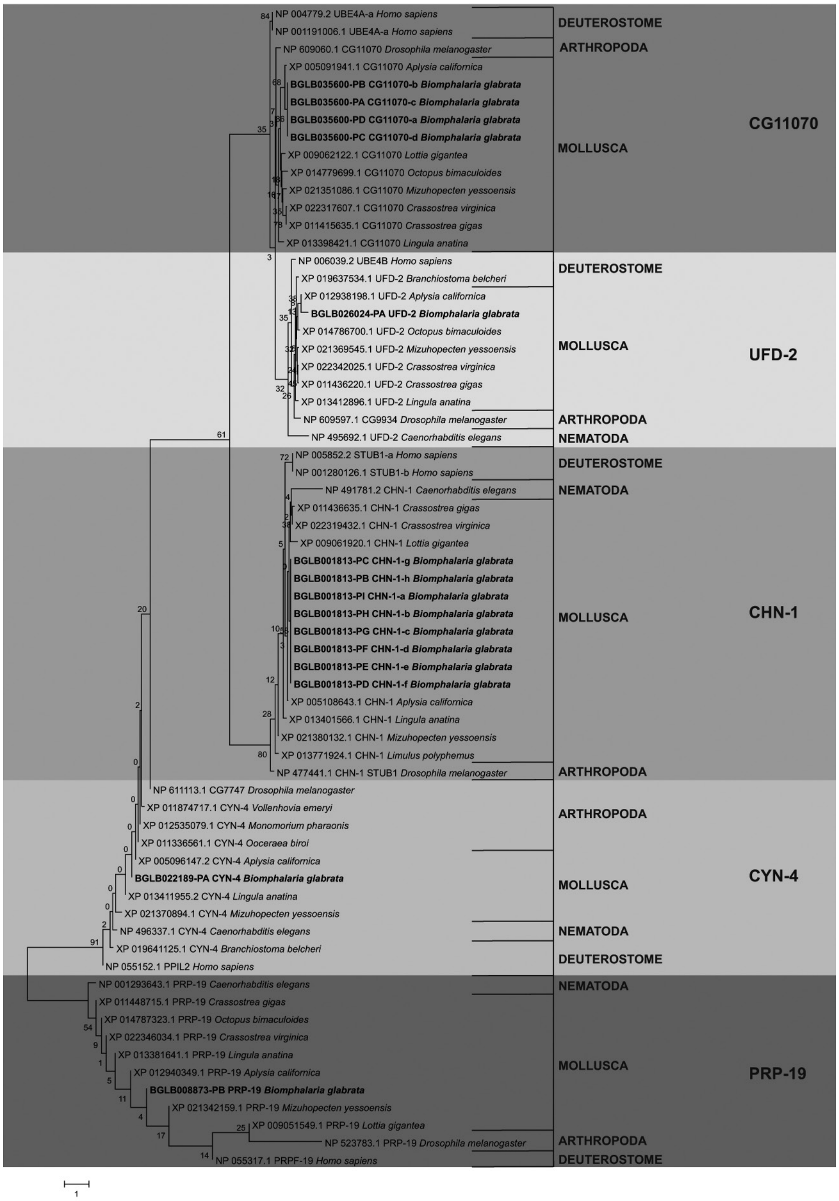

Fig. 8: evolutionary distribution of E3-U-box sequences identified in *Biomphalaria glabrata* and their orthologous organisms and model organisms.

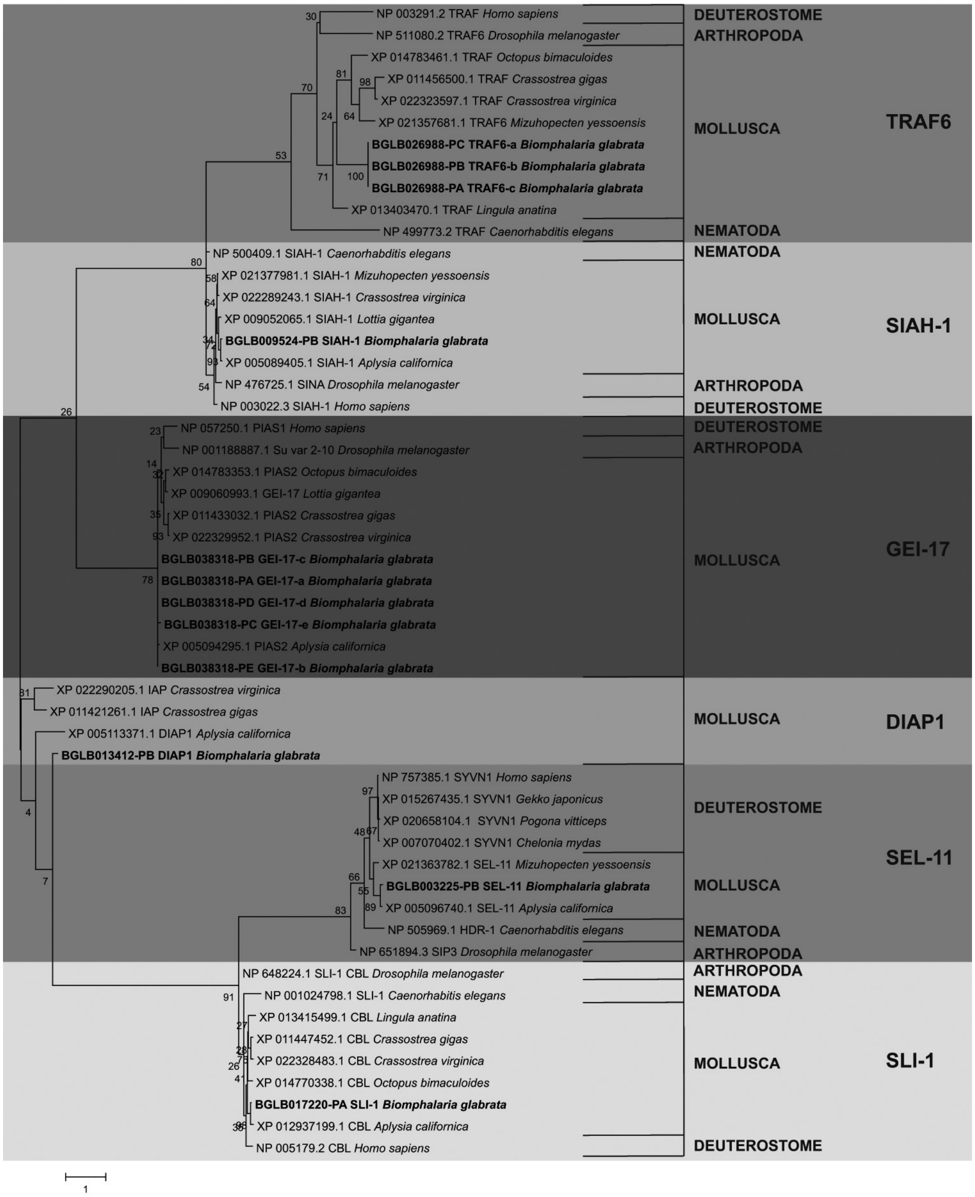

Fig. 9: phylogenetic tree representing the distribution found for the E3-RING finger sequences found in the data of *Biomphalaria glabrata* against orthologous organisms.

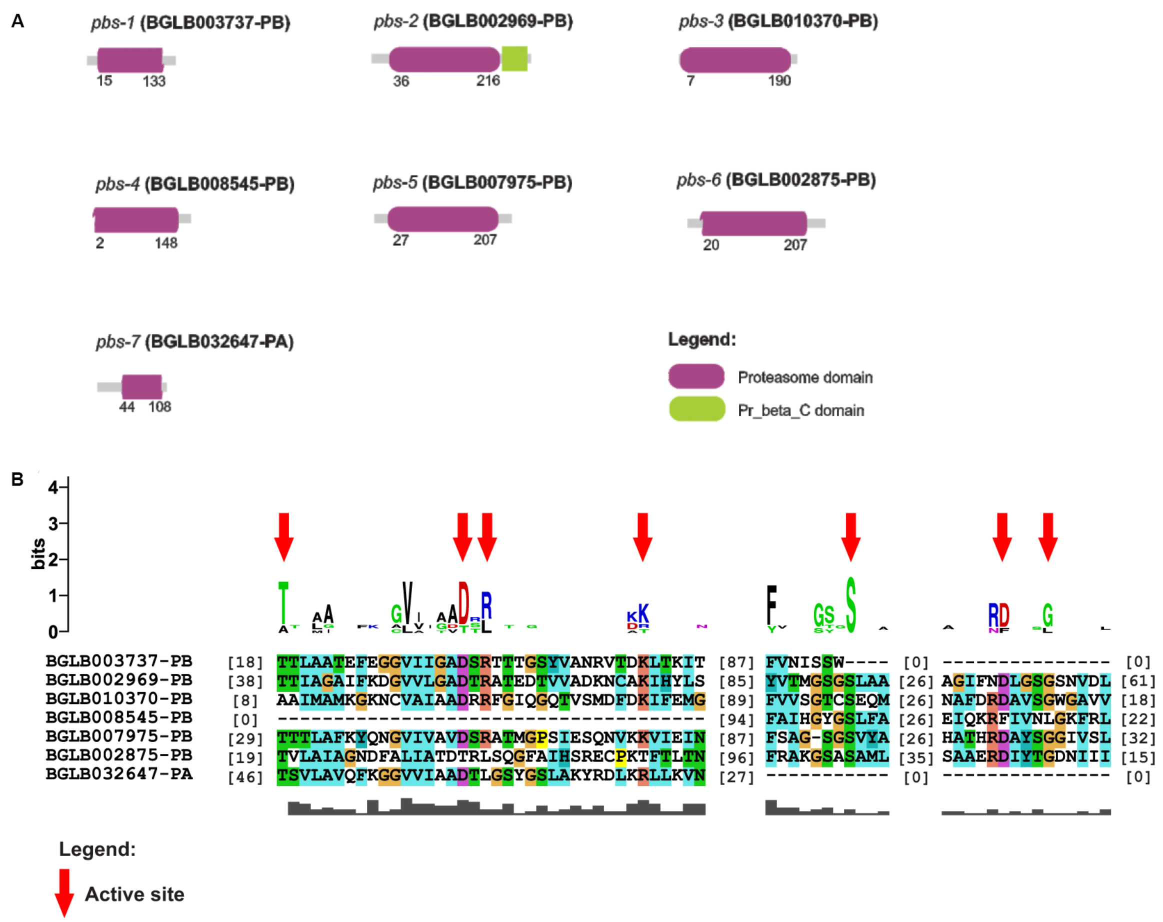

Fig. 10: (A) location and distribution of the proteasome domain identified in the sequences of the PBS group of *Biomphalaria glabrata*. (B) Active site found in the sequences participating in the PBS group of the *Biomphalaria glabrata* data.

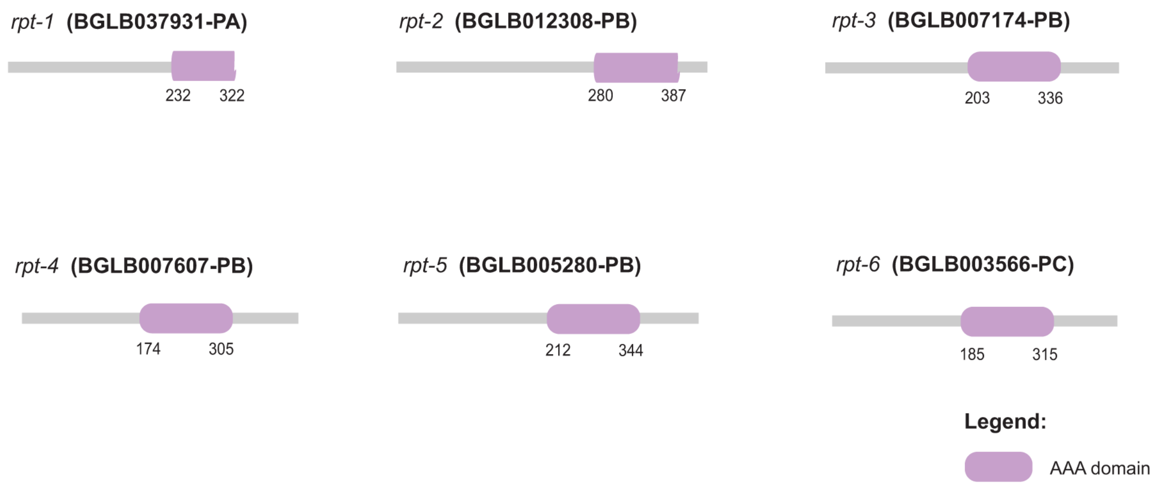

Fig. 11: position and distribution of the AAA domain in the sequences of *Biomphalaria glabrata* identified as RPT.

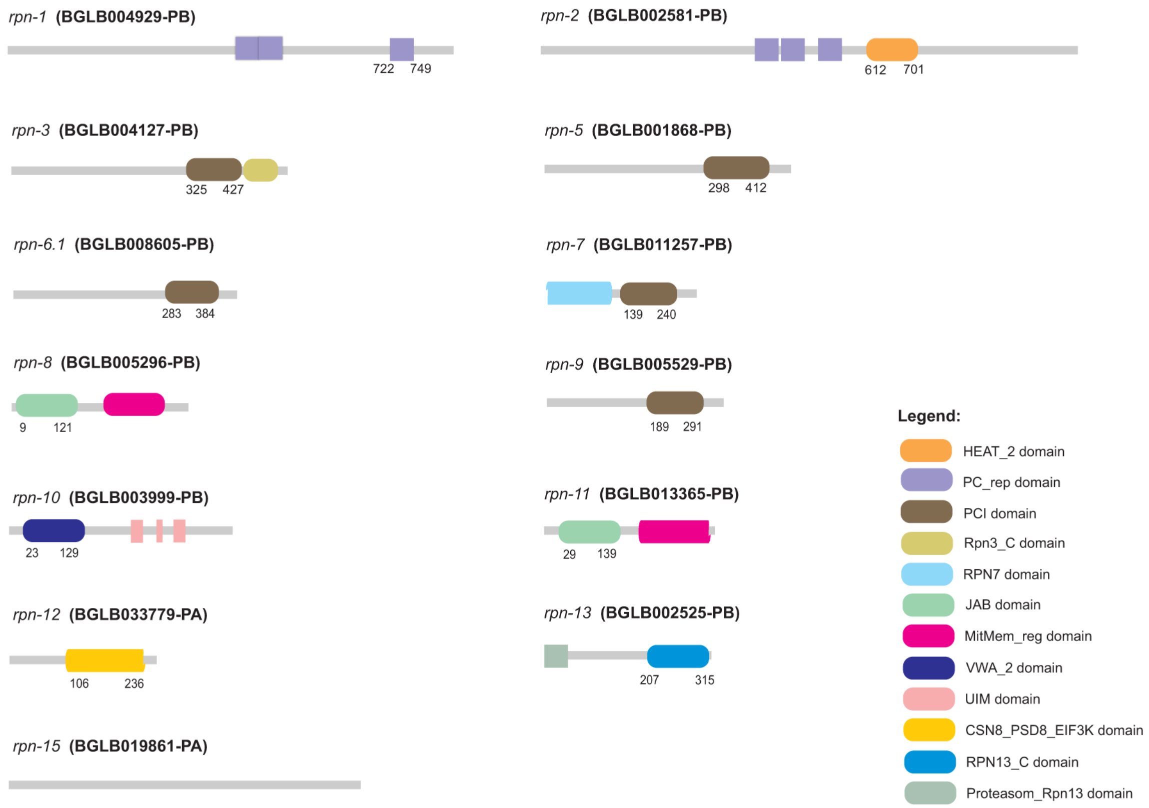

Fig. 12: presence and distribution of the conserved domains found in the sequences of *Biomphalaria glabrata* identified as RPN.

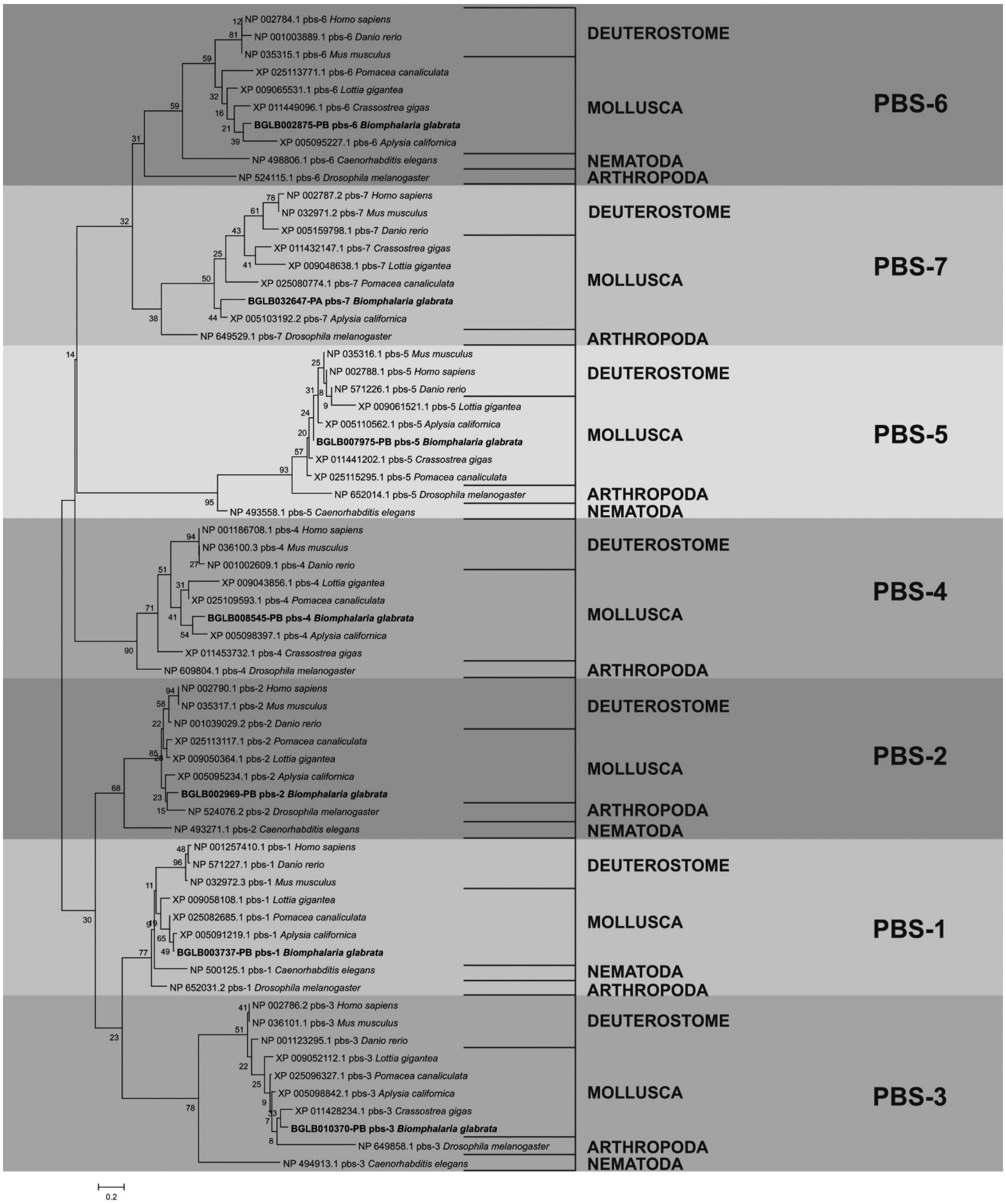

Fig. 13: phylogenetic tree generated from the PBS sequences identified in *Biomphalaria glabrata* and its orthologs.

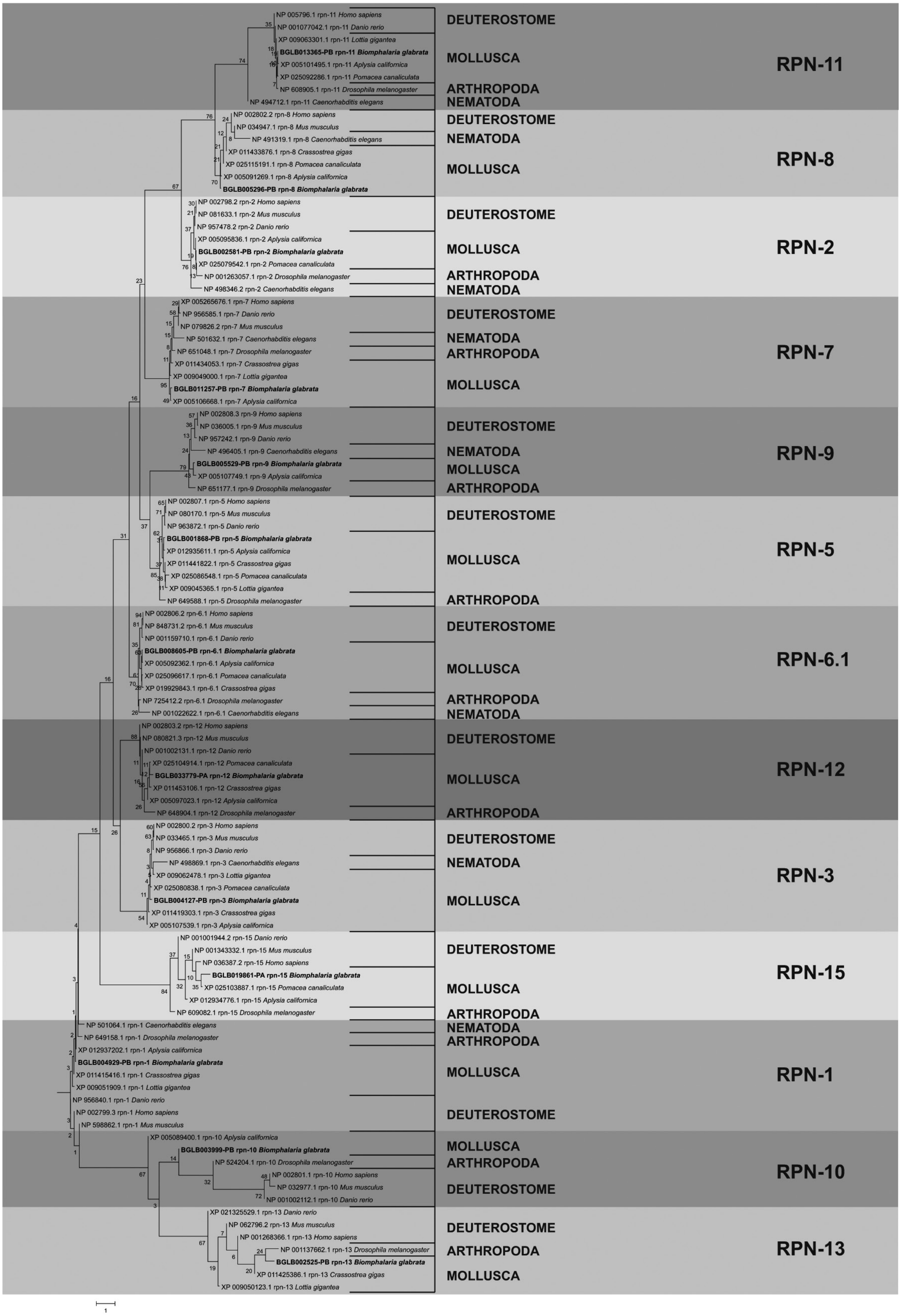

Fig. 14: representation of the evolutionary distribution of RPN sequences for *Biomphalaria glabrata* against their orthologous organisms.

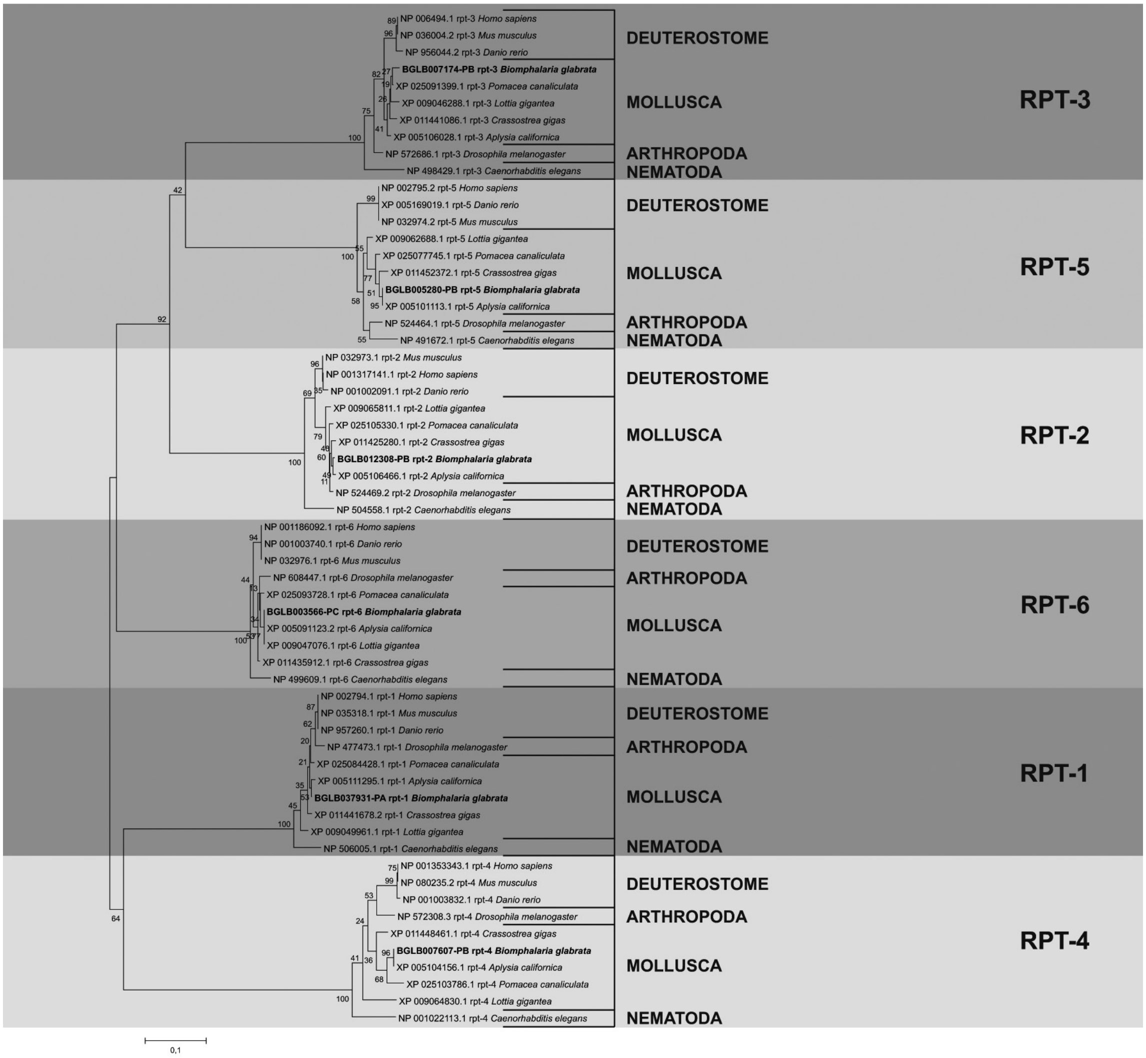

Fig. 15: evolutionary distribution of RPT sequences identified in *Biomphalaria glabrata*, their orthologous organisms and model organisms.
